# Effects of transcutaneous electrical nerve stimulation and manual laryngeal therapy on muscle tension dysphonia: randomized clinical trial

**DOI:** 10.1590/2317-1782/e20250264en

**Published:** 2026-06-19

**Authors:** Isadora de Oliveira Lemos, Kelly Cristina Alves Silvério, Mauriceia Cassol

**Affiliations:** 1 Programa de Pós-graduação em Ciências da Reabilitação, Universidade Federal de Ciências da Saúde, Porto Alegre, RS, Brasil.; 2 Departamento de Fonoaudiologia, Faculdade de Odontologia de Bauru – FOB, Universidade de São Paulo – USP, Bauru, SP, Brasil.

**Keywords:** Voice, Dysphonia, Muscle Tension, Speech Therapy, Transcutaneous Electric Nerve Stimulation, Manual Therapy

## Abstract

**Purpose:**

To compare the immediate effects of Transcutaneous Electrical Nerve Stimulation (TENS), Laryngeal Manual Therapy (LMT), and the combined application of both in women with Muscle Tension Dysphonia (MTD), considering vocal symptoms, voice quality, aerodynamic measures, and levels of perceived muscle tension on palpation.

**Methods:**

This was a randomized clinical trial including women with a speech-language diagnosis of MTD, allocated into three intervention groups: TENS, LMT, and TENS+LMT. Participants were assessed for vocal symptoms, musculoskeletal pain intensity, perceptual-auditory evaluation of voice quality using the GRBAS scale, maximum phonation times (MPT), S/Z ratio, and levels of perceived muscle tension on palpation using the Laryngeal Palpatory Scale (LPS).

**Results:**

Significant reductions in vocal symptoms, including vocal fatigue, and musculoskeletal pain were observed after the interventions, along with increased MPT in all groups. In the TENS group, breathiness decreased; in the LMT group, there was a significant reduction in overall voice alteration; and in the TENS+LMT group, reductions were observed in overall voice alteration and vocal tension. The LPS also showed a significant decrease in perceived muscle tension during phonation in the TENS+LMT group.

**Conclusion:**

All interventions showed positive effects, with significant reductions in vocal symptoms and musculoskeletal pain, as well as increases in MPT. The combined techniques were able to provide better vocal adjustments and reduce perceived muscle tension on palpation, demonstrating that both approaches were effective in promoting vocal and musculoskeletal benefits.

## INTRODUCTION

Efficient vocal production depends on a complex system involving the muscular action of structures in the vocal tract^(1,2)^. When the balance of this mechanism is altered due to excessive tension in the perilaryngeal musculature, changes in vocal quality and/or vocal complaints may arise, characterizing Muscle Tension Dysphonia (MTD)^(2-4)^.

MTD can manifest in various ways such as increased tension in the laryngeal muscles and cervical region^(1)^; complaints of musculoskeletal pain in the shoulder, neck, back and face^(5)^; symptoms related to the clinical picture of vocal fatigue such as tiredness when speaking, discomfort in the larynx and/or neck when using the voice, worsening of the voice at the end of the day, among others^(6)^; changes in vocal quality such as roughness, breathiness, compressed and/or tense voice^(1-3)^; laryngeal imaging signs such as hyperadduction of vocal folds, entry of ventricular bands during phonation, laryngeal compensatory mechanisms, glottal gaps during phonation and the presence of organofunctional lesions^(3,6,7)^.

Treatment of Muscle Tension Dysphonia (MTD) primarily involves reducing excessive muscle tension to promote balanced and effortless phonation, and may vary depending on the underlying cause identified in each patient ^(1-4)^. The literature describes the effectiveness of indirect approaches aimed at promoting better awareness of the mechanisms involved in vocal hyperfunction and direct approaches with techniques for relaxing the perilaryngeal muscles and promoting balanced and fluid phonation^(8-9)^.

Regarding techniques for relaxing the perilaryngeal region, laryngeal massages^(10-11)^, especially Laryngeal Manual Therapy (LMT)^(10)^, and the application of Transcutaneous Electrical Nerve Stimulation (TENS)^(12-13)^ can be highlighted.

LMT consists of massage with pressure and stretching movements of the muscles in the perilaryngeal region, in addition to laryngeal manipulation with movements of lowering and lateralization^(10)^. The main objective of this technique is to relax excessively tense muscles to promote balanced phonation, reposition the larynx in the neck and mobilize structures that are rigid and have reduced range of motion^(11,14)^. Studies have shown evidence that such movements cause changes in vocal quality, mainly with regard to tension^(15-16)^.

The application of TENS has also been cited in the literature as an effective tool in the treatment of hyperfunctional dysphonia. This technique promotes relaxation of the perilaryngeal and cervical region, assisting in phonatory balance^(13,17-24)^. The use of this resource involves the application of percutaneous electrodes to provide excitation of nerve fibers through an electrical current in the form of symmetrical or asymmetrical biphasic waves^(13)^. TENS can be applied at low^(18-20)^ or high frequency^(17-18)^, weak (stimulation at the sensory or sensorimotor threshold) or strong intensity (stimulation at the motor threshold) and in different stimulation fields, defined by the choice of electrode placement. Such adjustments depend on the therapeutic objective of the application. Studies point to different stimulation fields in the application of TENS for hyperfunctional dysphonia related to muscle tension. One example is the use of low-frequency, high-intensity TENS at the motor threshold, with electrodes in the suprahyoid region and upper muscle fibers of the trapezius muscles^(18-21)^. Another example is the application of low-frequency, low-intensity TENS at the motor threshold, with electrodes on the sides of the thyroid cartilage in the infrahyoid region, and in upper muscle fibers of the trapezius muscles^(22-23)^, with or without the association of vocal exercises^(22-24)^.

Due to the characteristic of excessive tension in the extrinsic laryngeal muscles of individuals with MTD, verifying the effectiveness of muscle and vocal rehabilitation methods in this population is of great clinical and scientific importance. Therefore, the objective of this study was to verify the effects of TENS, LMT, and the two techniques combined in women with MTD and to compare their effectiveness in relation to vocal quality, vocal symptoms, musculoskeletal pain complaints, muscle tension levels, and aerodynamic measurements.

## METHOD

### Study design

This is a randomized clinical trial. The study followed the CONSORT recommendations^(25)^. The clinical trial was registered on The Brazilian Registry of Clinical Trials (REBEC) platform under UTN number: U1111-1229-251. It was also approved by the Research Ethics Committee of the Proposing Institution under opinion 86530718.7.0000.5345.

### Participants

Recruitment was carried out with participants attending the Speech-language pathology service, linked to the Santa Casa de Misericórdia Hospital Complex in Porto Alegre, referred to the voice phonotherapy outpatient clinic. The sample consisted of women who met the inclusion criteria and agreed to participate in the study. The sample size calculation was based on the study by Silverio et al.^(18)^, considering the estimated population of 54 women attended at the reference service during the data collection period (finite population) and adopting a proportion of improvement in vocal quality of 50% between the groups (60% of one group shows results in better vocal quality before the intervention and 10% of the other group shows this result) and a minimum power of 80%, the estimate was at least 10 participants per group, based on a 95% confidence level for the interval estimates. All participants signed the Informed Consent Form to participate in the research study.

The study included female participants aged 18 to 55 years who presented with self-reported vocal complaints. The presence of vocal complaints was considered when the participant reported voice changes that interfered with their communication or well-being. The diagnosis of MTD was established based on a combination of clinical findings: presence of muscle tension in the perilaryngeal region, verified by the Laryngeal Palpatory Scale (LPS) protocol ^(26)^, associated with the presence of vocal tension identified using the GRBAS auditory-perceptual scale. The diagnosis of laryngeal pathologies, when present, was used only for sample characterization and was not an exclusion criterion. Participants with cardiac comorbidities, thyroid disorders, neurological diagnoses, a history of laryngeal surgery, as well as those who were undergoing speech therapy prior to or concurrent with the study, were excluded from the research.

The diagnosis of MTD was made by a speech-language pathologist specializing in voice and with more than five years of clinical experience in the field. The identification of the MTD clinical picture occurred through muscle palpation examination, in which the participant had to present a level of muscle tension in the perilaryngeal region, starting from the mild degree (other degrees were considered), according to the LPS protocol^(26)^. The presence of vocal tension in the auditory-perceptual evaluation, at least of mild degree, according to the GRBAS scale^(27)^. The diagnosis of laryngeal pathologies was concluded after a videolaryngoscopy examination performed by the otolaryngology team of the reference service. During the evaluation, participants were questioned about the use of their voice in their daily activities. Those who reported using their voice as their main work tool were classified as voice professionals, according to widely accepted criteria for this characterization in scientific and clinical contexts.

### Randomization

Randomization was performed using a randomized numbered list generated by the website “random.org”^(28)^. A researcher, who was not involved in the other stages of the study, allocated the participants to the intervention groups according to their order of arrival. This distribution was carried out after the speech-language pathology diagnosis of MTD was performed.

#### Assessment procedures

The primary outcome was vocal quality, consisting of auditory-perceptual voice analysis. Secondary outcomes were assessments of self-perception of vocal symptoms, vocal fatigue, and musculoskeletal pain, performed using self-perception protocols; aerodynamic measures, assessed by Maximum Phonation Times and s/z ratio; and muscle tension levels in the perilaryngeal region, assessed by applying the muscle palpation protocol^(26)^.

#### Assessment of vocal quality – auditory-perceptual analysis

For the auditory-perceptual analysis, voice recordings were made by emitting the sustained vowels [a] and [i] and counting from 1 to 10. Participants were instructed to perform the phonatory tasks at their usual speaking frequency. The tasks were performed with the participant standing, feet flat on the floor and spine erect, in a quiet environment. The emissions were recorded directly on a Sony Digital Voice Recorder ICD-PX240 and captured with a Karsect HT-9 headset microphone positioned 4 cm from the speaker's mouth.

Subsequently, the voices were randomized and sent to three blinded evaluators for the treatment groups and intervention time points. The evaluators analyzed the vocal quality of the participants using the GRBAS protocol^(27)^, scoring the following parameters: overall degree of vocal deviation, roughness, breathiness, and vocal tension. The asthenia parameter was not considered as it was a sample composed of participants with vocal tension. The evaluators assessed each parameter as follows: 0 – no deviation, 1 – slight deviation, 2 – moderate deviation, 3 – severe deviation. The percentages of inter-rater agreement for each parameter were: G: 94.6%; R: 91.1%; B: 91.1%; S: 92.8%. To verify the level of intra-rater agreement, 20% of the sample of voices chosen randomly by drawing lots were repeated. The percentages of intra-rater agreement for each evaluator were: evaluator 1: 92.2%; Evaluator 2: 93.8%; Evaluator 3: 92.2%. To assess inter- and intra-rater reliability, the weighted kappa agreement coefficient was used in conjunction with the 95% confidence interval.

#### Self-perception of vocal symptoms (VSS)

Participants were asked to complete the Vocal Symptoms Scale (VSS) protocol^(29)^ consisting of 30 questions that map the individual's vocal symptoms. Responses were scored according to a Likert-type scale: zero – never, one – almost never, two – sometimes, three – almost always, and four – always. The total score, which can range from 0 to 120 points, was considered for analysis.

#### Self-perception of vocal fatigue (VFI)

Vocal Fatigue symptoms were assessed using the Brazilian version of the Vocal Fatigue Index (VFI) protocol^(30)^. The protocol has nineteen questions scored on a Likert-type scale: zero – never, one – almost never, two – sometimes, three – almost always and four – always, according to the occurrence of the symptom. The total score, which can range from 0 to 76 points, was considered for the analysis. The version by Zambon et al.^30^ was used, as data collection began before the publication of the protocol in the validated and translated version for Brazilian Portuguese.

#### Maximum phonation times and s/z relationship

The maximum phonation times (MPT) of the vowels /a/, /e/, /i/, /o/ and /u/ and the sounds /s/ and /z/ were timed and considered in seconds ^(31-32)^. Participants were instructed to maintain an orthostatic position with their arms extended along their bodies during the emissions. After performing a maximum inspiration, participants were instructed to produce phonation during maximum expiration, to ensure a measurement with maximum phonatory and respiratory efficiency. Two emissions of each phoneme were performed, and the longest times emitted in each vowel and in the phonemes /s/ and /z/ were considered. The timing was performed using an iPhone SE (Apple).

#### Musculoskeletal pain

The assessment of musculoskeletal pain was performed using a protocol based on the Nordic Musculoskeletal Symptom Questionnaire – NMSQ^(33)^. This questionnaire relates musculoskeletal pain to the body part being investigated and to the intensity of the pain reported. For this study, the following regions were selected, according to a greater relationship with the clinical picture of MTD: upper back, lower back, temporal region, masseter, submandibular region, larynx, anterior and posterior neck region^(5)^. Pain intensity was analyzed using an analog scale with a length of 100 mm, in which the participant marked with a vertical line the point where she reported her pain level. The left edge meant no pain and the right edge, the worst possible pain.

#### Assessment of muscle tension level

The assessment of the level of muscle tension sensation was carried out using the Laryngeal Palpatory Scale muscle palpation protocol^(26)^. At the time of application in the study, the Laryngeal Palpation Scale (LPS) protocol did not have a validated Brazilian version. For this study, the instrument was translated and adapted for clinical application by the authors, maintaining the same original assessment parameters. This protocol has observational and muscle palpation parameters that verify the muscular conditions of perilaryngeal structures, which influence the level of muscle tension sensation during phonation. The assessment was performed by a speech-language pathologist, postgraduate in the area of ​​voice, with more than five years of experience in applying the protocol and with clinical experience in the area of ​​voice. This measurement methodology was chosen according to a similar study^(34)^. The assessment of muscle palpation depends on the evaluator's experience, as there is anatomical variation in relation to the evaluator's fingers, differences related to the touch performed on the study participant and the configuration of the neck evaluated^(35)^. Muscle palpation measurements from the protocol were performed with a softer touch (tenderness) and a firmer touch (tightness), both at rest and during the phonatory tasks described for each parameter, as per the protocol description. In this study, only the muscle palpation parameters were considered for the total score. Muscle tension levels with a soft touch (tenderness) were also analyzed separately at rest and during the phonatory tasks of sustained /i/ vowel emission and counting from 1 to 10. Only the soft touch was chosen because it is similar to the type of touch used in Laryngeal Manual Therapy, which was used in this study. In addition, the parameters of high laryngeal position in the neck and resistance to laryngeal lateralization were analyzed.

### Intervention procedures

#### Intervention groups

The research intervention groups were: TENS, LMT, and TENS+LMT (association of TENS and LMT, in this order of application). Therapies in the three groups were performed twice a week for a period of six weeks, totaling twelve individual sessions^(18-20)^. Pre-treatment assessments were performed one week before the first session, and post-treatment assessments were performed one week after the last therapy session. During the proposed intervention, participants did not receive guidance regarding vocal hygiene nor were they instructed to perform exercises at home.

#### Electrotherapy (TENS)

Electrotherapy was applied according to the study by Silverio et al.^(18)^. The equipment used in the application was the IBRAMED brand, Neurodyn Portable model. The silicone-carbon electrodes (3.0 cm x 4.0 cm) were fixed in the submandibular region and in the trapezius muscle, bilaterally. The placement of electrodes in the trapezius muscle depended on the therapist's palpation and verification of the region with greater muscle tension. The electrodes were positioned perpendicularly to the muscle fibers of this region. In the submandibular region, the electrodes were fixed in the region just below the chin, taking care that the electrodes did not reach the laryngeal region and the bony part of the mandible. After fixing the electrodes, the participant was placed in a supine position, on a stretcher prepared for the application of electrotherapy.

The participant was instructed to maintain a comfortable posture. The TENS application lasted 25 minutes, with five minutes of adaptation to the stimulus and 20 minutes of stimulation. The parameters used were: low-frequency TENS, with a symmetrical biphasic square pulse, device adjusted with a pulse width T: 200ms and a frequency of 10 Hz. To begin electrotherapy, the therapist adjusted the stimulus intensity to 5mA (milliamperes) for the first three minutes and increased it according to the participant's comfort threshold. In the final ten minutes, the therapist maintained the highest intensity at the comfort threshold, as indicated by the participant herself, so that stimulation at the motor threshold, at a strong intensity, was possible.

#### Laryngeal manual therapy (LMT)

LMT was applied for 24 minutes. The therapist was positioned behind the participant and began massaging the sternocleidomastoid, suprahyoid, and laryngeal muscles bilaterally, with downward circular movements, kneading, and stretching in each muscle group, in addition to laryngeal displacement. During the procedure, the participant was instructed to remain silent and relaxed^(10)^. In the present study, the application of three minutes of massage to the sternocleidomastoid muscles was considered; three minutes of massage in the suprahyoid region; one minute and thirty seconds of application of gliding and lowering movement in the laryngeal region. It continued with one minute and thirty seconds of application of lateral displacement movement of the thyroid region; and repetition of three minutes of massage to the sternocleidomastoid muscles. The sequence adopted was based on a previous study^(36)^. This sequence was performed twice to match the application time of TENS.

#### Electrotherapy (TENS) followed by laryngeal manual therapy (LMT)

Subjects included in this group participated in the two interventions sequentially and in the same order. First, TENS was applied, but for twelve minutes, with two minutes of adaptation to the stimulus and ten minutes of stimulation. Laryngeal Manual Therapy was applied next, also for twelve minutes. Three minutes of massage was applied to the sternocleidomastoid muscles; three minutes of massage in the suprahyoid region; one minute and thirty seconds of gliding and lowering movement in the larynx region; and one minute and thirty seconds of lateral displacement movement of the thyroid region; repetition of three minutes of massage in the sternocleidomastoid muscles^(36)^.

### Statistical analysis

Quantitative variables were described by mean and standard deviation/standard error, and categorical variables by absolute and relative frequencies. The Shapiro-Wilk normality test was applied to verify the distribution of the variables.

To compare means between groups, Analysis of Variance (ANOVA) was applied. For the comparison of proportions, Pearson's chi-square test, along with analysis of adjusted residuals, was used.

The comparison between time points and between groups was performed using the Generalized Estimating Equations (GEE) model complemented by the Least Significant Difference (LSD) test. The linear model was used for variables with a normal distribution, and the Tweedie model with a logarithmic function was applied for variables with an asymmetrical distribution. All results were adjusted for variables that presented p<0.10 in the group comparison analysis (voice professional and laryngeal diagnoses).

The significance level adopted was 5% (p≤0.05) and the analyses were performed using SPSS version 27.0.

## RESULTS

**Figure 1:**
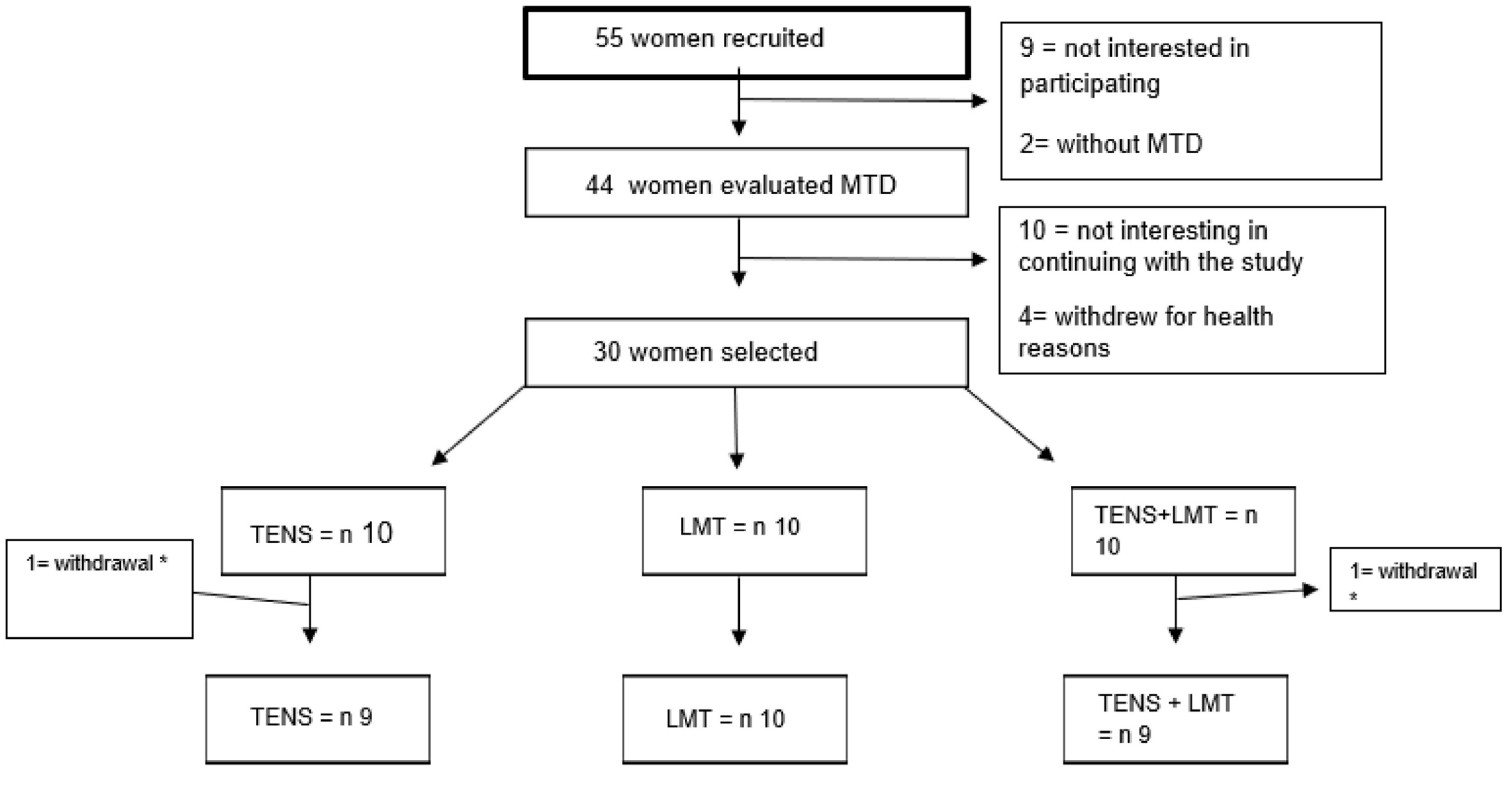
Flowchart of research participants

According to Figure 1, 44 women were included in the study, of whom 28 completed all stages of the study. The participants were distributed into the groups according to the randomization performed in the research study.

*Withdrawal: Participants who interrupted the study before the completion of the planned interventions, regardless of the reason

According to Table 1, which characterizes the sample, there was no statistically significant difference between the groups regarding the average age of the participants. A statistically significant difference was found between the groups regarding the use of professional voice and laryngeal pathologies.

**Table 1:** Characterization of the studied groups, according to age, profession and laryngeal characteristics

**Variables**	**TENS** **(n=9)**	**LMT (n=10)**	**TENS+LMT (n=9)**	**P-value**
Age (years) – mean ± SD	40.7 ± 8.1	41.9 ± 11.1	41.8 ± 12.0	0.963
Voice professional – n (%)				0.001
Yes	6 (66.7)	0 (0.0)	7 (77.8)^*^	
No	3 (33.3)	10 (100)*	2 (22.2)	
Laryngeal diagnosis – n(%)				0.099
No change	2 (22.2)	8 (80.0)	3 (33.3)	
Vocal cyst	1 (11.1)	2 (20.0)	1 (11.1)	
Vocal nodules	3 (33.3)	0 (0.0)	3 (33.3)	
Vocal gap	3 (33.3)	0 (0.0)	2 (22.2)	

* Statistically significant association by the adjusted residuals test at a 5% significance level

Regarding the auditory-perceptual evaluation, as shown in Table 2, there was an improvement in the overall vocal quality parameter in both the LMT group and the TENS+LMT group. The TENS group did not show a statistically significant difference. No statistically significant change was found in the roughness parameter in the evaluated groups. However, in the breathiness parameter, a statistically significant decrease was detected in the TENS group. There was an increase in the TENS+LMT group, with a statistically significant difference observed in the comparison between groups. Regarding vocal tension, only the TENS+LMT group showed a statistically significant improvement.

**Table 2:** Auditory-perceptual analysis with intragroup comparison (before and after therapeutic interventions) and between the studied groups: TENS, LMT, and TENS+LMT adjusted for voice professionals and laryngeal diagnoses

**Variables**	**TENS**	**LMT**	**TENS+LMT**	**p -value**
	**Mean ± SE**	**Mean ± SE**	**Mean ± SE**	
G				
Pre	1.94 ± 0.20	1.72 ± 0.16	1.63 ± 0.26	0.764
Post	1.48 ± 0.18	1.30 ± 0.14	1.27 ± 0.15	0.406
Pre-Post Difference	0.45 (-0.03 a 0.94)	0.42 (0.10 a 0.74)	0.35 (0.02 a 0.69)	0.972
p -value	0.066	0.011	0.038	
R				
Pre	1.56 ± 0.17	1.44 ± 0.12	1.68 ± 0.25	0.416
Post	1.26 ± 0.17	1.24 ± 0.08	1.19 ± 0.18	0.754
Pre-Post Difference	0.29 (-0.22 a 0.81)	0.20 (-0.05 a 0.46)	0.50 (-0.04 a 1.03)	0.640
p -value	0.264	0.113	0.068	
B				
Pre	1.23 ± 0.41	0.45 ± 0.24	0.22 ± 0.14	0.083
Post	0.46 ± 0.19^ab^	0.10 ± 0.09^b^	0.66 ± 0.22^a^	0.015
Pre-Post Difference	0.77 (0.14 a 1.41)^b^	0.35 (-0.11 a 0.81)^b^	-0.44 (-0.88 a 0.00)^a^	0.007
p -value	0.017	0.134	0.051	
S				
Pre	1.36 ± 0.17	1.50 ± 0.19	1.33 ± 0.16	0.857
Post	1.14 ± 0.19	1.30 ± 0.17	0.89 ± 0.19	0.353
Pre-Post Difference	0.22 (-0.20 a 0.64)	0.20 (-0.05 a 0.46)	0.46 (0.12 a 0.79)	0.433
p -value	0.310	0.112	0.007	

a,b,c Identical letters do not differ by the Least Significant Difference (LSD) test at a 5% significance level

Table 3 demonstrated a statistically significant increase in Maximum Phonation Time (MPT) after intervention in all evaluated groups; no statistically significant difference was found between them. In the Vocal Symptom Scale (VSS) and the Vocal Fatigue Index (VFI) protocol, all groups significantly reduced their scores after intervention. However, no statistically significant difference was detected between them. The S/Z Index did not show a statistically significant change.

**Table 3:** Comparison of aerodynamic measurements and vocal symptoms within groups (before and after therapeutic interventions) and between the studied groups: TENS, LMT, and TENS+LMT adjusted for voice professionals and laryngeal diagnoses

**Variables**	**TENS**	**LMT**	**TENS+LMT**	**p -value**
	**Mean ± SE**	**Mean ± SE**	**Mean ± SE**	
MPT total				
Pre	7.81 ± 0.73	9.51 ± 1.39	8.96 ± 1.12	0.205
Post	10.8 ± 1.38	11.9 ± 1.56	11.4 ± 0.77	0.946
Pre-Post Difference	-2.95 (-5.19 a -0.71)	-2.42 (-3.95 a-0.89)	-2.41 (-4.16 a -0.66)	0.918
p -value	0.010	0.002	0.007	
S/Z Ratio				
Pre	1.12 ± 0.08	1.00 ± 0.05	1.14 ± 0.09	0.410
Post	0.99 ± 0.08	1.07 ± 0.08	1.05 ± 0.08	0.808
Pre-Post Difference	0.13 (-0.03 a 0.29)	-0.04 (-0.26 a 0.18)	0.09 (-0.13 a 0.31)	0.120
p -value	0.112	0.696	0.416	
VSS				
Pre	58.0 ± 8.38	55.8 ± 7.06	54.8 ± 3.66	0.629
Post	26.6 ± 5.76	20.3 ± 4.69	28.2 ± 4.81	0.579
Pre-Post Difference	31.4 (12.1 a 50.8)	35.5 (20.5 a 50.5)	26.6 (19.1 a 34.0)	0.553
p -value	0.001	<0.001	<0.001	
VFI				
Pre	45.5 ± 5.01	48.1 ± 3.98	44.5 ± 2.23	0.940
Post	23.9 ± 4.98	23.5 ± 3.88	24.2 ± 2.92	0.721
Pre-Post Difference	21.6 (10.9 a 32.2)	24.6 (16.2 a 33.0)	20.3 (14.4 a 26.2)	0.718
p -value	<0.001	<0.001	<0.001	

**Key:** MPT = Maximum Phonation Time; LMT = Laryngeal Manual Therapy; VSS = Vocal Symptom Scale; VFI = Vocal Fatigue Index

Regarding the intensity of musculoskeletal pain self-reported by the participants, as shown in Table 4, the evaluated regions of upper back, lower back, masseter muscle, and posterior neck showed a statistically significant decrease after the interventions in all groups. In the submandibular region, the TENS and LMT groups did not show a statistically significant change. In the anterior neck region, the LMT group showed no change in the analysis. In the larynx region, improvement was observed in the LMT and TENS+LMT groups, with a statistically significant difference when compared to the TENS group.

**Table 4:** Comparison of musculoskeletal pain in different body regions within groups (before and after therapeutic interventions) and between the groups studied: TENS, LMT, and TENS+LMT adjusted for voice professionals and laryngeal diagnoses

**Body Regions**	**TENS**	**LMT**	**TENS+LMT**	**p -value**
	**Mean ± SE**	**Mean ± SE**	**Mean ± SE**	
Upper back				
Pre	59.3 ± 8.96	50.9 ± 11.9	53.8 ± 10.8	0.609
Post	20.5 ± 7.56	17.9 ± 6.46	15.1 ± 4.47	0.773
Pre-Post Difference	38.8 (15.3 a 62.4)	33.0 (9.83 a 56.2)	38.7 (15.3 a 62.1)	0.895
p -value	0.001	0.005	0.001	
Lower back				
Pre	40.0 ± 9.18	48.2 ± 14.0	53.8 ± 10.8	0.319
Post	27.4 ± 9.57	16.2 ± 8.70	19.0 ± 5.94	0.255
Pre-Post Difference	12.6 (1.51 a 23.7)	32.1 (9.74 a 54.4)	34.9 (12.6 a 57.1)	0.131
p -value	0.026	0.005	0.002	
Temporal				
Pre	39.9 ± 16.2	61.8 ± 25.3	17.4 ± 5.62	0.061
Post	11.7 ± 4.52	5.76 ± 4.29	7.13 ± 4.92	0.756
Pre-Post Difference	28.2 (-1.80 a 58.2)	56.0 (8.21 a 103.8)	10.3 (-5.24 a 25.8)	0.323
p -value	0.065	0.022	0.194	
Masseter				
Pre	25.4 ± 7.78	45.6 ± 18.8	35.2 ± 8.93	0.200
Post	11.3 ± 5.12	12.8 ± 7.69	13.2 ± 5.48	0.608
Pre-Post Difference	14.1 (4.42 a 23.7)	32.7 (2.19 a 63.3)	22.0 (0.54 a 43.5)	0.714
p -value	0.004	0.036	0.044	
Submandibular				
Pre	30.4 ± 8.77	19.3 ± 8.64	35.0 ± 12.2	0.828
Post	10.6 ± 5.22	6.76 ± 5.53	9.76 ± 4.82	0.697
Pre-Post Difference	19.8 (-1.01 a 40.6)	12.5 (-7.17 a 32.2)	25.3 (1.22 a 49.3)	0.954
p -value	0.062	0.213	0.039	
Larynx				
Pre	31.4 ± 11.7	39.3 ± 12.8	51.5 ± 11.5	0.720
Post	19.5 ± 6.17	1.88 ± 1.35	12.0 ± 9.09	0.292
Pre-Post Difference	11.9 (-17.5 a 41.3)^a^	37.4 (12.9 a 62.0)^b^	39.5 (10.8 a 68.2)^b^	0.019
p -value	0.428	0.003	0.007	
Front of the neck				
Pre	32.5 ± 12.2	18.4 ± 8.82	42.6 ± 11.9	0.578
Post	4.36 ± 3.31	1.97 ± 1.68	11.5 ± 7.61	0.417
Pre-Post Difference	28.1 (5.43 a 50.8)	16.4 (-0.60 a 33.5)	31.1 (2.96 a 59.3)	0.684
p -value	0.015	0.059	0.030	
Posterior neck				
Pre	55.1 ± 8.81	60.7 ± 15.1	51.8 ± 10.2	0.581
Post	18.3 ± 5.89	22.6 ± 6.82	16.2 ± 5.93	0.781
Pre-Post Difference	36.8 (16.9 a 56.7)	38.2 (10.7 a 65.7)	35.6 (13.7 a 57.5)	0.938
p -value	<0.001	0.007	0.001	

a,b,c Identical letters do not differ by the Least Significant Difference (LSD) test at 5% significance

In Table 5, when analyzing the levels of muscle tension sensation upon palpation, a reduction was observed in all groups evaluated in the total score. During palpation at rest, a decrease in muscle tension sensation was observed in the submental region in the TENS and LMT groups; a decrease in the left infrahyoid region; a decrease in the right infrahyoid region except in the TENS+LMT group. The left cricothyroid showed a decrease except in the TENS group; a reduction in the right cricothyroid in all groups with a statistically significant difference between the groups; and the sternocleidomastoid muscle region showed a reduction on the left and right except in the LMT group. When analyzing the levels of muscle tension sensation during palpation during phonation, a reduction was observed in the submental region in all groups; the left and right infrahyoid regions showed a reduction in the LMT and TENS+LMT groups with a statistically significant difference between the groups; and the left cricothyroid region showed a significant reduction when comparing the groups. In the right cricothyroid region, improvement was observed in all groups; in the left sternocleidomastoid muscle region, all groups showed improvement. A statistically significant difference was observed between the groups; in the right sternocleidomastoid muscle region, no reduction was observed in the TENS group, and a significant difference was found when comparing the TENS group with the other two groups.

**Table 5:** Comparison of the level of muscle tension sensation in different body regions within groups (before and after therapeutic interventions) and between the groups studied: TENS, LMT, and TENS+LMT adjusted for voice professionals and laryngeal diagnoses

**Regions**	**TENS**	**LMT**	**TENS+LMT**	**p -value**	
	**Mean ± SE**	**Mean ± SE**	**Mean ± SE**		
Total					
Pre	61.5 ± 10.0	53.0 ± 7.31	60.0 ± 7.77	0.609	
Post	29.2 ± 6.56	14.9 ± 2.40	14.4 ± 2.22	0.052	
Pre-Post Difference	32.3 (23.0 a 41.6)^a^	38.1 (26.8 a 49.4)^ab^	45.7 (32.9 a 58.4)^b^	<0.001	
p -value	<0.001	<0.001	<0.001		
P.R.Submentum					
Pre	1.13 ± 0.33	0.84 ± 0.26	0.97 ± 0.31	0.858	
Post	0.42 ± 0.21	0.22 ± 0.14	0.37 ± 0.19	0.714	
Pre-Post Difference	0.71 (0.09 a 1.33)	0.62 (0.13 a 1.12)	0.60 (0.00 a 1.20)	0.874	
p -value	0.024	0.013	0.051		
P.R.Infrahyoid (L)					
Pre	0.95 ± 0.32	0.40 ± 0.14	1.12 ± 0.46	0.734	
Post	0.43 ± 0.21	0.11 ± 0.07	0.09 ± 0.08	0.377	
Pre-Post Difference	0.52 (0.13 a 0.92)	0.29 (0.02 a 0.56)	1.03 (0.08 a 1.98)	0.280	
p -value	0.010	0.038	0.034		
P.R.Infrahyoid (R)					
Pre	0.92 ± 0.29	0.66 ± 0.27	0.95 ± 0.50	0.921	
Post	0.52 ± 0.22	0.07 ± 0.07	0.09 ± 0.09	0.171	
Pre-Post Difference	0.40 (0.10 a 0.71)	0.59 (0.07 a 1.10)	0.86 (-0.15 a 1.87)	0.093	
p -value	0.009	0.026	0.096		
P.R.Cricothyroid (L)					
Pre	0.89 ± 0.31	0.90 ± 0.24	1.42 ± 0.41	0.232	
Post	0.41 ± 0.21	0.10 ± 0.10	0.00 ± 0.00	0.121	
Pre-Post Difference	0.48 (-0.28 a 1.24)	0.80 (0.32 a 1.28)	1.42 (0.61 a 2.24)	0.215	
p -value	0.218	0.001	<0.001		
P.R.Cricothyroid (R)					
Pre	0.99 ± 0.27	0.91 ± 0.26	1.77 ± 0.38	0.056	
Post	0.20 ± 0.12	0.00 ± 0.00	0.00 ± 0.00	0.266	
Pre-Post Difference	0.79 (0.21 a 1.37)^a^	0.91 (0.40 a 1.43)^ab^	1.77 (1.03 a 2.50)^b^	0.044	
p -value	0.008	<0.001	<0.001		
P.R. ECOM (L)					
Pre	1.06 ± 0.31	1.23 ± 0.62	0.75 ± 0.21	0.461	
Post	0.45 ± 0.17^b^	0.09 ± 0.09^ab^	0.07 ± 0.07^a^	0.019	
Pre-Post Difference	0.61 (0.27 a 0.95)	1.14 (-0.04 a 2.31)	0.68 (0.23 a 1.12)	0.072	
p -value	<0.001	0.058	0.003		
P.R. ECOM (R)					
Pre	0.94 ± 0.31	1.18 ± 0.59	0.85 ± 0.29	0.941	
Post	0.54 ± 0.21^b^	0.18 ± 0.12^ab^	0.07 ± 0.07^a^	0.010	
Pre-Post Difference	0.40 (0.02 a 0.79)	1.00 (-0.12 a 2.13)	0.77 (0.14 a 1.40)	0.066	
p -value	0.040	0.081	0.016		
P.P.Submentum					
Pre	1.79 ± 0.46	1.25 ± 0.33	1.25 ± 0.28	0.605	
Post	1.10 ± 0.34	0.34 ± 0.18	0.31 ± 0.14	0.118	
Pre-Post Difference	0.69 (0.10 a 1.29)	0.91 (0.39 a 1.43)	0.94 (0.33 a 1.54)	0.089	
p -value	0.022	<0.001	0.002		
P.P.Infrahyoid (L)					
Pre	1.13 ± 0.38	0.82 ± 0.30	1.32 ± 0.28		0.863
Post	1.03 ± 0.37^b^	0.27 ± 0.13^ab^	0.09 ± 0.09^a^		0.024
Pre-Post Difference	0.11 (-0.09 a 0.30)^a^	0.55 (0.00 a 1.10)^ab^	1.23 (0.64 a 1.81)^b^		0.005
p -value	0.288	0.050	<0.001		
P.P.Infrahyoid (R)					
Pre	1.30 ± 0.33	1.09 ± 0.32	1.30 ± 0.30		0.880
Post	1.08 ± 0.29^b^	0.17 ± 0.12^a^	0.20 ± 0.12^a^		0.001
Pre-Post Difference	0.22 (-0.06 a 0.50)^a^	0.92 (0.37 a 1.47)^b^	1.09 (0.48 a 1.71)^b^		0.001
p -value	0.126	0.001	<0.001		
P.P.Cricothyroid (L)					
Pre	1.13 ± 0.35	1.96 ± 0.72	1.13 ± 0.29		0.711
Post	0.58 ± 0.19^b^	0.30 ± 0.20^ab^	0.08 ± 0.07^a^		0.005
Pre-Post Difference	0.55 (0.08 a 1.02)^a^	1.66 (0.33 a 3.00)^ab^	1.06 (0.44 a 1.67)^b^		0.038
p -value	0.021	0.014	<0.001		
P.P.Cricothyroid (R)					
Pre	1.34 ± 0.38	1.36 ± 0.53	1.53 ± 0.41		0.836
Post	0.54 ± 0.20	0.00 ± 0.00	0.19 ± 0.12		0.067
Pre-Post Difference	0.79 (0.34 a 1.25)	1.36 (0.32 a 2.41)	1.34 (0.52 a 2.17)		0.092
p -value	<0.001	0.010	0.001		
P.P. ECOM (L)					
Pre	1.38 ± 0.44	1.47 ± 0.54	1.58 ± 0.33		0.766
Post	0.63 ± 0.21^b^	0.10 ± 0.10^a^	0.10 ± 0.09^a^		0.048
Pre-Post Difference	0.75 (0.16 a 1.33)^a^	1.37 (0.35 a 2.39)^b^	1.48 (0.81 a 2.16)^b^		0.020
p -value	0.012	0.009	<0.001		
P.P. ECOM (R)					
Pre	1.18 ± 0.37	2.16 ± 0.86	1.21 ± 0.27		0.691
Post	0.65 ± 0.20^b^	0.12 ± 0.11^a^	0.07 ± 0.06^a^		0.003
Pre-Post Difference	0.52 (0.00 a 1.05)^a^	2.05 (0.40 a 3.70)^b^	1.14 (0.59 a 1.69)^b^		0.006
p -value	0.052	0.015	<0.001		
High larynx position					
Pre	1.55 ± 0.25^b^	1.69 ± 0.38^ab^	1.00 ± 0.20^a^		0.031
Post	0.57 ± 0.14	0.53 ± 0.17	0.36 ± 0.13		0.171
Pre-Post Difference	0.98 (0.56 a 1.40)	1.17 (0.31 a 2.02)	0.63 (0.12 a 1.14)		0.938
p -value	<0.001	0.007	0.016		
Lateralization of the larynx					
Pre	1.82 ± 0.22	2.12 ± 0.41	1.66 ± 0.28		0.917
Post	0.85 ± 0.25	0.72 ± 0.22	0.65 ± 0.16		0.576
Pre-Post Difference	0.97 (0.54 a 1.39)	1.40 (0.68 a 2.13)	1.01 (0.62 a 1.40)		0.671
p -value	<0.001	<0.001	<0.001		

a,b,c Identical letters do not differ by the Least Significant Difference (LSD) test at a 5% significance level

**Caption:** P.R. = Palpation at rest; P.P. = Palpation during phonation; ECOM = sternocleidomastoid muscle

Only palpation with a gentle touch (“tenderness”) was considered for analysis.

Regarding the parameters of high laryngeal position in the neck and resistance to laryngeal lateralization, there was a significant decrease in all groups after the interventions, with no difference between them.

## DISCUSSION

The techniques applied in this study have been analyzed as possible resources in the rehabilitation of individuals with dysphonia associated with muscle tension^(15-21,37)^. This study compared the techniques individually and in combination to verify their effects in relation to vocal quality parameters, vocal self-perception and musculoskeletal pain, aerodynamic measures and muscle tension levels.

Regarding the characterization of the sample and groups, there was a predominance of middle-aged participants, voice professionals, and those presenting laryngeal alterations of functional and organofunctional origin, which is consistent with the profile of participants with MTD^(3-4)^. In the LMT group, specific differences were observed regarding the professional use of the voice and laryngeal alterations, considering that the sample was predominantly composed of non-voice professionals and, for the most part, without laryngeal alterations. This sample characteristic was carefully controlled and considered in the statistical analyses in order to minimize biases and ensure the validity of the results. Women in this age group who use their voice professionally are more susceptible to developing dysphonia, as they present biological and behavioral factors that favor an imbalance in laryngeal muscle function^(38)^. There is a difference in proportion between the female and male larynx, as well as the fact that female vocal folds are shorter and less thick, which increases the risk of collisions and the difficulty of absorbing lesions. There is also an asymmetrical distribution of hyaluronic acid in the lamina propria of female vocal folds, which makes glottal closure difficult^(39-41)^. Women are also more susceptible to psychosocial factors that cause emotional problems^(1)^. This increases the risk of developing stress and anxiety, leading to excessive muscle tension and chronic musculoskeletal pain^(33)^. When these women use their voice professionally, the risk of developing dysphonia increases, as many of them do not have adequate knowledge about vocal health and exhibit inappropriate vocal behavior^(41)^.

This study demonstrated the positive effects of the two techniques, applied in isolation and in combination, in relation to vocal symptoms and vocal fatigue. The women reported improvement in their vocal symptoms, which positively affects their daily lives in a functional, physical and emotional way. Previous studies indicate that the use of both techniques used in this research study has positive effects on the self-reported symptoms of the participants^(18,21)^. In addition, there was a significant decrease in symptoms of vocal fatigue reported by the women in the study. Vocal fatigue can affect participants with MTD, as it is related to inadequate vocal and muscular adjustments and a feeling of effort when speaking^(42)^.

Regarding self-perception of musculoskeletal pain, all three groups showed positive responses regarding the reduction of self-reported pain in the perilaryngeal and cervical regions. These results corroborate studies that have demonstrated a decrease in pain symptoms in the perilaryngeal and adjacent regions with the use of TENS and LMT^(13,18,22)^. Regarding the structures evaluated, the improvement in the larynx region reported by participants in the LMT and TENS+LMT groups, who received LMT in the laryngeal region, stands out compared to the TENS group, which received TENS application in isolation. It is possible to conclude that, by using direct manipulation of the extrinsic laryngeal musculature, LMT was more effective in reducing pain sensation in this region^(11)^. Based on the results found, it can be inferred that the combination of electrotherapy techniques associated with laryngeal manual therapy demonstrates more positive results in the neck region and laryngeal framework. This is because the application of the associated techniques provided a greater feeling of relaxation and muscle comfort in the perilaryngeal and cervical region^(10-11)^.

The increase in Maximum Phonation Times (MPT) stands out in the three groups evaluated. MPT is a clinical parameter that indicates the ability to control the aerodynamic and myoelastic forces of the larynx^(43)^. The significant increase observed in the present study may be related to a decrease in myoelastic action in the balance between the two forces, which may have provided improved muscle and respiratory function during phonation^(44)^. The s/z ratio showed normality before the techniques were applied and remained so after therapy, which may indicate that the measurement was not able to indicate the hyperfunctional pattern of the participants evaluated.

Regarding the assessment of vocal quality, it was possible to verify an improvement in the overall degree of vocal deviation in the groups that received isolated LMT (LMT) or associated with TENS (TENS+LMT). It can be inferred that the use of LMT had a better effect on phonatory balance, improving the vocal quality of women in these two groups. This result differs from another study^(21)^, which found better immediate effects on vocal quality in women with behavioral dysphonia who used TENS, compared to those who received only LMT. The relationship between vocal tension parameters and breathiness is noteworthy. A similar relationship was found in another study^(24)^ with the application of low-frequency TENS associated with vocal exercises. In the TENS and LMT groups there was little reduction in vocal tension. In the TENS+LMT group, however, the significant reduction in tension suggests an increase in breathiness, which may have occurred due to a probable adjustment of the larynx. Another study^(45)^ applied LMT to participants with primary MTD and concluded that acoustic and glottic closure measures for several participants revealed insufficient glottic closure after treatment, when hyperfunction was reduced with LMT. The authors conclude that, for these participants, the application of complementary techniques to assist in balanced glottic closure function is important.

Regarding the levels of muscle tension sensation upon palpation, the analyzed groups showed significant improvement after the interventions, particularly the group that used electrotherapy techniques combined with laryngeal manual therapy. The applied techniques were effective in reducing the sensation of muscle tension in the extrinsic laryngeal muscles upon palpation and in adjacent structures, as their main effect is to increase the impression of muscle relaxation in these structures^(13,18)^. The TENS+LMT group, by combining the two techniques, had a better effect in reducing the sensation of muscle tension upon palpation in this region, especially when evaluated during phonation. The combined techniques more precisely promoted the sensation of reduced tension in the extrinsic laryngeal muscles, which may have been caused by a decrease in the activation of the muscle compensation system during the participants' phonation. This may have had a positive impact on the functioning of this muscle group.

Among the limitations of this study, the absence of a control group stands out, which restricted the possibilities for comparing the results obtained. Furthermore, a discrepancy was observed between the groups regarding professional voice use and the presence of vocal alterations, a factor considered in the statistical analyses. As the allocation of participants was performed by randomization, it was expected that the groups would present homogeneous characteristics. However, in studies with limited sample size, differences between groups can occur due to chance. To minimize this potential bias, multivariate analysis was performed adjusting for the variables "voice professional" and "laryngeal diagnosis," with the aim of making the groups more comparable and ensuring greater validity in the interpretation of the results. In addition, it was not possible to perform acoustic voice analysis or laryngeal imaging after the interventions due to the lack of adequate laboratory infrastructure to guarantee the necessary methodological rigor. Therefore, it is recommended that future research include acoustic voice analysis and laryngeal imaging exams to deepen the understanding of the effects of the applied techniques. It is also suggested that complementary assessments, such as surface electromyography, be used to objectively investigate the impact of the interventions on the perilaryngeal musculature.

## CONCLUSION

The findings of this study demonstrate that the proposed interventions exerted relevant therapeutic effects in women with Muscle Tension Dysphonia. There was a reduction in vocal symptoms, a decrease in vocal fatigue, a reduction in musculoskeletal pain, and an increase in maximum phonation times in the three groups evaluated. Regarding vocal quality, the TENS and LMT techniques alone decreased the degrees of breathiness and the overall degree of dysphonia, respectively. However, only when applied in combination were they able to decrease the overall degree of dysphonia and the degree of vocal tension, providing better vocal adjustment, since vocal tension is an important characteristic of the studied population. Regarding muscle tension, only LMT alone or combined with TENS was able to reduce the sensation of muscle tension upon palpation. Therefore, the combined techniques were able to provide better vocal adjustments and decrease the sensation of muscle tension upon palpation.
