# Supplementary material for: Effects of transcutaneous electrical nerve stimulation and manual laryngeal therapy on muscle tension dysphonia: randomized clinical trial
Source: Codas. 2026 Jun 19;38(4):e20250264. doi: 10.1590/2317-1782/e20250264en (PMC13322494; doi:10.1590/2317-1782/e20250264en)
Supplement: Translation

# Translation: Efeitos da estimulação elétrica nervosa transcutânea e da terapia manual laríngea na disfonia por tensão muscular: ensaio clínico randomizado

**DOI:** 10.1590/2317-1782/e20250264pt

**Authors:** Isadora de Oliveira Lemos, Kelly Cristina Alves Silvério, Mauriceia Cassol

**Affiliations:** 1 Programa de Pós-graduação em Ciências da Reabilitação, Universidade Federal de Ciências da Saúde, Porto Alegre, RS, Brasil.; 2 Departamento de Fonoaudiologia, Faculdade de Odontologia de Bauru – FOB, Universidade de São Paulo – USP, Bauru, SP, Brasil.

**Keywords:** Voz, Disfonia, Dor Musculoesquelética, Terapia Manual, Estimulação Elétrica Nervosa Transcutânea, Fonoaudiologia

## Abstract

**Objetivo:**

Comparar os efeitos imediatos da Estimulação Elétrica Nervosa Transcutânea (TENS), da Terapia Manual Laríngea (TML) e da aplicação combinada de ambas em mulheres com Disfonia por Tensão Muscular (DTM), considerando sintomas vocais, qualidade vocal, medidas aerodinâmicas e níveis de sensação de tensão muscular à palpação.

**Método:**

Trata-se de um ensaio clínico randomizado. Foram incluídas mulheres com diagnóstico fonoaudiológico de Disfonia por Tensão Muscular, alocadas em três grupos de intervenção: TENS, TML e TENS+TML. As participantes foram avaliadas quanto aos sintomas vocais, intensidade da dor musculoesquelética, avaliação perceptivo-auditiva da qualidade vocal pela escala GRBAS, tempos máximos de fonação (TMF), relação S/Z e níveis de sensação de tensão muscular à palpação com o protocolo Laryngeal Palpatory Scale (LPS).

**Resultados:**

Observou-se redução significativa dos sintomas vocais, incluindo fadiga vocal, e de dor musculoesquelética após as intervenções, bem como aumento dos TMF em todos os grupos. No grupo TENS, houve redução da soprosidade vocal; no grupo TML, observou-se redução significativa do grau geral da alteração vocal; e no grupo TENS+TML, uma redução do grau geral de alteração e da tensão vocal. O LPS demonstrou diminuição significativa da sensação de tensão muscular à fonação no grupo TENS+TML.

**Conclusão:**

Todas as intervenções apresentaram efeitos positivos, com redução significativa dos sintomas vocais e da dor musculoesquelética, além do aumento dos tempos máximos de fonação. As técnicas combinadas foram capazes de proporcionar melhores ajustes vocais e diminuir a sensação de tensão muscular à palpação, evidenciando que ambas as abordagens foram eficazes em promover benefícios vocais e musculoesqueléticos.

## INTRODUÇÃO

Uma produção vocal eficiente depende de um complexo sistema que envolve a ação muscular de estruturas do trato vocal^(1,2)^. Quando o equilíbrio desse mecanismo é alterado devido à tensão excessiva na musculatura perilaríngea, podem surgir alterações na qualidade vocal e/ou queixas vocais, caracterizando a Disfonia por Tensão Muscular (DTM)^(2-4)^.

A DTM pode se manifestar de diversas formas como aumento da tensão na musculatura laríngea e na região cervical^(1)^; queixas de dor musculoesquelética em região de ombros, pescoço, costas e face^(5)^; sintomas relacionados ao quadro clínico de fadiga vocal como cansaço ao falar, desconforto na laringe e/ou no pescoço ao usar a voz, piora da voz ao final do dia, entre outros^(6)^; alterações de qualidade vocal como rugosidade, soprosidade, voz comprimida e/ou tensa^(1-3)^; sinais de imagem laríngea como hiperadução de pregas vocais, entrada de bandas ventriculares à fonação, mecanismos compensatórios laríngeos, fendas glóticas à fonação e presença de lesões organofuncionais^(3,6,7)^.

O tratamento da Disfonia por Tensão Muscular (DTM) envolve, principalmente, a diminuição da tensão muscular excessiva para promover uma fonação equilibrada e sem esforço, podendo variar conforme a causa subjacente identificada em cada paciente ^(1-4)^. A literatura descreve a eficácia de abordagens indiretas com objetivo de promover uma melhor conscientização acerca dos mecanismos que envolvem a hiperfunção vocal e de abordagens diretas com técnicas para relaxamento da musculatura perilaríngea e para promover uma fonação equilibrada e fluida^(8-9)^.

Quanto às técnicas para relaxamento da região perilaríngea, é possível destacar as massagens laríngeas^(10-11)^, em especial a Terapia Manual Laríngea (TML)^(10)^, e a aplicação de Estimulação Elétrica Nervosa Transcutânea (TENS)^(12-13)^.

A TML consiste na massagem com movimentos de pressão e alongamento de músculos da região perilaríngea, além da manipulação laríngea com presença de movimentos de abaixamento e lateralização da mesma^(10)^. O principal objetivo desta técnica é relaxar a musculatura excessivamente tensa para promover uma fonação equilibrada, reposicionar a laringe no pescoço e mobilizar estruturas que estão rígidas e com diminuição de amplitude de movimento^(11,14)^. Estudos têm mostrado evidências que tais movimentos provocam mudanças na qualidade vocal, principalmente no que diz respeito à tensão^(15-16)^.

A aplicação da TENS também tem sido citada pela literatura como uma ferramenta eficaz no tratamento de disfonias hiperfuncionais. Esta técnica promove o relaxamento da região perilaríngea e cervical, auxiliando no equilíbrio fonatório^(13,17-24)^. O uso deste recurso envolve a aplicação de eletrodos percutâneos para propiciar a excitação de fibras nervosas por meio de uma corrente elétrica em forma de ondas bifásicas simétricas ou assimétricas^(13)^. A TENS pode ser aplicada em baixa^(18-20)^ ou alta frequência^(17-18),^ fraca (estímulo no limiar sensorial ou sensório-motor) ou forte intensidade (estímulo no limiar motor) e em diferentes campos de estimulação, definidos pela escolha na colocação dos eletrodos. Tais ajustes dependem do objetivo terapêutico da aplicação. Estudos apontam diferentes campos de estimulação na aplicação da TENS para disfonias hiperfuncionais relacionadas à tensão muscular. Um exemplo é o uso da TENS de baixa frequência e forte intensidade no limiar motor, com eletrodos na região supra-hióidea e fibras musculares superiores dos músculos trapézios^(18-21)^. Outro exemplo, é a aplicação de TENS de baixa frequência e fraca intensidade no limiar motor, com eletrodos nas laterais da cartilagem tireoide em região infra-hióidea, e em fibras musculares superiores dos músculos trapézios^(22-23)^, com ou sem associação de exercícios vocais^(22-24)^.

Devido à característica de tensão excessiva da musculatura extrínseca da laringe de indivíduos com DTM, a verificação da eficácia de métodos de reabilitação muscular e vocal nesta população é de grande importância clínica e científica. Desta forma, o objetivo deste estudo foi verificar os efeitos da TENS, da TML e das duas técnicas associadas em mulheres com DTM e comparar as suas eficácias em relação à qualidade vocal, sintomas vocais, às queixas de dor musculoesquelética, aos níveis de tensão muscular, às medidas aerodinâmicas.

## MÉTODOS

### Desenho do estudo

Trata-se de um ensaio clínico randomizado. O estudo seguiu as recomendações do CONSORT^(25)^. O ensaio clínico foi registrado na plataforma The Brazilian Registry of Clinical Trials (REBEC) sob o número de UTN: U1111-1229-251. Também foi aprovado no Comitê de Ética em Pesquisa da Instituição Proponente sob o parecer 86530718.7.0000.5345.

### Participantes

O recrutamento foi realizado com as participantes atendidas no serviço de Fonoaudiologia, vinculado ao Complexo Hospitalar da Santa Casa de Misericórdia de Porto Alegre, encaminhadas ao ambulatório de fonoterapia da voz. A amostra foi composta pelas mulheres que preencheram os critérios de inclusão e que concordaram participar do estudo. O cálculo amostral foi baseado no estudo de Silverio et al.^(18)^, considerando a população estimada de 54 mulheres atendidas no serviço de referência no período de coleta (população finita) e adotando-se a proporção de melhora na qualidade vocal de 50% entre os grupos (60% de um grupo apresenta resultados na qualidade vocal melhor antes da intervenção e 10% do outro grupo apresenta esse resultado) e poder mínimo de 80%, a estimativa foi de pelo menos 10 participantes por grupo, tomando como base nível de confiança de 95% para as estimativas intervalares. Todas as participantes assinaram o Termo de Consentimento Livre e Esclarecido para participação da pesquisa.

Foram incluídas no estudo participantes do sexo feminino, com idade entre 18 e 55 anos, que apresentavam queixa vocal autorreferida. A presença de queixa vocal foi considerada quando a participante relatava alterações na voz que interferiam em sua comunicação ou bem-estar. O diagnóstico de DTM foi estabelecido a partir da combinação de achados clínicos: presença de tensão muscular na região perilaríngea, verificada pelo protocolo Laryngeal Palpatory Scale (LPS)^(26)^, associada à presença de tensão vocal identificada por meio da escala perceptivo-auditiva GRBAS. O diagnóstico de patologias laríngeas, quando presente, foi utilizado apenas para caracterização da amostra, não sendo critério de exclusão. Foram excluídas da pesquisa participantes com comorbidades cardíacas, distúrbios tireoidianos, diagnóstico neurológico, histórico de cirurgia laríngea, bem como aquelas que estivessem em acompanhamento fonoaudiológico anterior ou simultâneo ao estudo.

O diagnóstico de DTM foi realizado por uma fonoaudióloga especialista em voz e com mais de cinco anos de experiência clínica na área. A identificação do quadro clínico de DTM ocorreu por meio de exame de palpação muscular, em que a participante deveria apresentar nível de tensão muscular na região perilaríngea, a partir do grau leve (outros graus foram considerados), de acordo com o protocolo LPS^(26)^. A presença de tensão vocal na avaliação perceptivo-auditiva, no mínimo de grau leve, de acordo com a escala GRBAS^(27)^. O diagnóstico de patologias laríngeas foi concluído após exame de videolaringoscopia realizado pela equipe de otorrinolaringologistas do serviço de referência. As participantes foram questionadas, durante a avaliação, sobre o uso da voz em suas atividades diárias. Foram classificadas como profissionais da voz aquelas que referiram utilizar a voz como principal instrumento de trabalho, de acordo com critérios amplamente aceitos para essa caracterização em contextos científicos e clínicos.

### Randomização

A randomização foi realizada por meio de uma lista numérica randomizada gerada pelo site “random.org”^(28)^. Um pesquisador, que não estava envolvido com as demais etapas do estudo, realizou a alocação das participantes nos grupos de intervenção, de acordo com a ordem de chegada. Esta distribuição foi realizada após a realização do diagnóstico fonoaudiológico de DTM.

### Procedimentos de avaliação

O desfecho primário foi a qualidade vocal, composta pela análise perceptivo-auditiva da voz. Os desfechos secundários foram as avaliações de autopercepção de sintomas vocais, de fadiga vocal e de dor musculoesquelética, realizadas pela aplicação de protocolos de autopercepção; as medidas aerodinâmicas, avaliadas pelos Tempos Máximos de Fonação e relação s/z; e os níveis de tensão muscular da região perilaríngea, avaliados pela aplicação do protocolo de palpação muscular^(26)^.

#### Avaliação da qualidade vocal – análise perceptivo-auditiva

Para a análise perceptivo-auditiva foram realizadas gravações da voz por meio da emissão das vogais [a] e [i] sustentadas e de contagem de 1 a 10. As participantes foram orientadas a realizarem as tarefas fonatórias na frequência habitual de fala. As tarefas foram executadas com a participante em pé, com os pés apoiados no chão e com a coluna ereta, em ambiente silencioso. As emissões foram registradas diretamente em um gravador da marca Sony Digital Voice Recorder ICD-PX240 e captadas com o microfone modelo headset Karsect HT-9 posicionado a 4 cm da boca do falante.

Posteriormente, as vozes foram randomizadas e enviadas a três avaliadores cegados para os grupos de tratamento e momentos de intervenção. Os avaliadores analisaram a qualidade vocal das participantes por meio do protocolo GRBAS^(27)^, pontuando os seguintes parâmetros: grau geral de desvio vocal, rugosidade, soprosidade e tensão vocal. O parâmetro astenia não foi considerado por se tratar de uma amostra composta por participantes com presença de tensão vocal. Os avaliadores avaliaram cada parâmetro da seguinte forma: 0 – sem desvio, 1- desvio discreto, 2- desvio moderado, 3- desvio intenso. Os percentuais de nível de concordância inter-avaliadores para cada parâmetro foram: G: 94,6%; R: 91,1%; B: 91,1%; S: 92,8%. Para a verificação do nível de concordância intra-avaliadores, foram repetidas 20% da amostra de vozes escolhidas de forma aleatória por meio de sorteio. Os percentuais de nível de concordância intra-avaliadores para cada avaliador foram: avaliador 1: 92,2%; avaliador 2: 93,8%; avaliador 3: 92,2%. Para avaliar a confiabilidade interavaliadores e intraavaliadores foi utilizado o coeficiente de concordância kappa ponderado em conjunto com o intervalo de 95% de confiança.

#### Autopercepção de sintomas vocais (ESV)

Solicitou-se às participantes que respondessem o protocolo Escala de Sintomas Vocais (ESV)^(29)^ composto por 30 questões que mapeiam os sintomas vocais do indivíduo. As respostas foram pontuadas de acordo com uma escala do tipo Likert: zero – nunca, um – quase nunca, dois – às vezes, três – quase sempre e quatro – sempre. Foi considerado para a análise o escore total, que pode variar de 0 a 120 pontos.

#### Autopercepção de fadiga vocal (IFV)

Os sintomas de Fadiga Vocal foram avaliados por meio da versão brasileira do protocolo Vocal Fatigue Index (VFI)^(30)^. O protocolo possui dezenove questões pontuadas em uma escala do tipo Likert: zero – nunca, um – quase nunca, dois – às vezes, três – quase sempre e quatro – sempre, conforme ocorrência do sintoma. Foi considerado para a análise o escore total, que pode variar de 0 a 76 pontos. Foi utilizada a versão de Zambon et al.^30^, pois a coleta de dados iniciou em momento anterior ao da publicação do protocolo na versão validada e traduzida para o português brasileiro.

#### Tempos máximos de fonação e relação s/z

Os tempos máximos de fonação (TMF) das vogais /a/, /e/, /i/, /o/ e /u/ e dos sons /s/ e /z/ foram cronometrados e considerados em segundos(s)^(31-32)^. As participantes foram orientadas a manterem-se em posição ortostática e com os braços estendidos ao longo do corpo durante as emissões. Após realizarem uma inspiração máxima, as participantes foram instruídas a produzirem a fonação em expiração máxima, para garantirem uma medida com máxima eficiência fonatória e respiratória. Foram realizadas duas emissões de cada fonema e foram considerados os maiores tempos emitidos em cada vogal e nos fonemas /s/ e /z/. A cronometragem foi realizada por meio de aparelho Iphone SE (Apple).

#### Dor musculoesquelética

A avaliação de dor musculoesquelética foi realizada por meio de um protocolo baseado no Questionário de Sintomas Musculoesquelético Nórdico–NMSQ^(33)^. Este questionário relaciona a dor musculoesquelética com a parte do corpo que está sendo investigada e com a intensidade da dor referida. Para este estudo, foram selecionadas as seguintes regiões, de acordo com uma maior relação com o quadro clínico de DTM: parte superior das costas, parte inferior das costas, região temporal, masseter, região submandibular, laringe, região anterior e posterior do pescoço^(5)^. A intensidade da dor foi analisada por meio de uma escala analógica com comprimento de 100 mm, em que a participante marcou com um traço vertical o ponto em que referia seu nível de dor. A borda esquerda significou nenhuma dor e a borda direita, à pior dor possível.

#### Avaliação do nível de tensão muscular

A avaliação do nível de sensação de tensão muscular deu-se por meio do protocolo de palpação muscular *Laryngeal Palpatory Scale* ^(26)^. No momento da aplicação na pesquisa, o protocolo *Laryngeal Palpation Scale* (LPS) não possuía versão brasileira validada. Para esta pesquisa, o instrumento foi traduzido e adaptado para aplicação clínica pelas autoras, mantendo-se os mesmos parâmetros originais de avaliação. Este protocolo possui parâmetros observacionais e de palpação muscular que verificam as condições musculares de estruturas perilaríngeas, que influenciam no nível de sensação de tensão muscular à fonação. A avaliação foi realizada por uma fonoaudióloga, pós-graduada na área de voz, com mais de cinco anos de experiência na aplicação do protocolo e com experiência clínica na área de voz. Esta metodologia de aferição foi escolhida de acordo com um estudo semelhante^(34)^. A avaliação de palpação muscular depende da experiência do avaliador, pois existe variação anatômica com relação aos dedos do avaliador, diferenciações relacionadas ao toque realizado no participante do estudo e à configuração do pescoço avaliado^(35)^. Medidas de palpação muscular do protocolo foram realizadas com toque mais suave *(tenderness)* e com toque mais firme *(tightness)* e foram realizadas em repouso e durante as tarefas fonatórias descritas em cada parâmetro, conforme a descrição do protocolo. No presente estudo, para o escore total foram considerados apenas os parâmetros de palpação muscular. Também foram analisados separadamente os níveis de tensão muscular com toque suave *(tenderness)* em repouso e durante as tarefas fonatórias de emissão da vogal /i/ sustentada e contagem de 1 a 10. Optou-se por utilizar apenas o toque suave, pois é o tipo de toque semelhante ao utilizado na Terapia Manual Laríngea, utilizada no estudo. Além disso, foram analisados os parâmetros de posição alta da laringe no pescoço e de resistência à lateralização da laringe.

### Procedimentos de intervenção

#### Grupos de intervenção

Os grupos de intervenção da pesquisa foram: TENS; TML e TENS+TML (associação da TENS e da TML, nesta ordem de aplicação). As terapias nos três grupos foram realizadas com a frequência de duas vezes por semana por um período de seis semanas, totalizando doze sessões individuais^(18-20)^. As avaliações pré-tratamento foram realizadas uma semana antes da primeira sessão, e as avaliações pós-tratamento foram realizadas uma semana após a última sessão de terapia. Durante a intervenção proposta, os participantes não receberam orientações relativas à higiene vocal nem foram instruídos a realizar exercícios em domicílio.

#### Eletroterapia (TENS)

A eletroterapia foi aplicada de acordo com o estudo de Silverio et al.^(18)^. O equipamento utilizado na aplicação foi da marca IBRAMED modelo *Neurodyn Portable*. Os eletrodos de silicone-carbono (3,0 cm x 4,0 cm) foram fixados na região submandibular e no músculo trapézio, bilateralmente. A colocação de eletrodos no músculo trapézio dependeu da palpação do terapeuta e da verificação de região com maior tensão muscular. Os eletrodos foram posicionados perpendicularmente às fibras musculares dessa região. Já na região submandibular, os eletrodos foram fixados na região logo abaixo do queixo, cuidando para que os eletrodos não atingissem a região laríngea e a parte óssea da mandíbula. Após a fixação dos eletrodos, a participante foi colocada em posição decúbito dorsal, em maca preparada para a aplicação da eletroterapia.

A participante foi orientada a manter-se em postura confortável. A aplicação da TENS durou 25 minutos, sendo cinco minutos de adaptação ao estímulo e 20 minutos de estimulação. Os parâmetros utilizados foram: TENS de baixa frequência, com pulso quadrático bifásico simétrico, aparelho ajustado com largura de pulso *T*: 200ms e frequência 10 Hz. Para iniciar a eletroterapia, o terapeuta ajustou a intensidade do estímulo em 5mA (miliamperes) nos primeiros três minutos e foi aumentando conforme o limiar de conforto da participante. Nos dez minutos finais, o terapeuta manteve a maior intensidade do limiar de conforto, referenciada pela própria participante, de forma que fosse possível uma estimulação no limiar motor, em forte intensidade.

#### Terapia manual laríngea (TML)

A TML foi aplicada por 24 minutos. O terapeuta ficou posicionado atrás da participante e iniciou a massagem nos músculos esternocleidomastóideos, suprahióideos e na região da laringe, bilateralmente, com movimentos descendentes circulares, amassamento e alongamento em cada grupo muscular, além do deslocamento da laringe. Durante o procedimento, a participante foi instruída a ficar em silêncio e relaxada^(10)^. No presente estudo foi considerada a aplicação de três minutos de massagem nos músculos esternocleidomastóideos; três minutos de massagem na região suprahióidea; um minuto e trinta segundos de aplicação de movimento de deslizamento e abaixamento na região da laringe; e um minuto e trinta segundos de aplicação de movimento de deslocamento lateral da região da tireoide; repetição de três minutos de massagem nos músculos esternocleidomastóideos. A sequência adotada foi baseada em um estudo prévio^(36)^. Esta sequência foi realizada duas vezes para equiparar com o tempo de aplicação da TENS.

#### Eletroterapia (TENS) seguida da terapia manual laríngea (TML)

Os sujeitos incluídos neste grupo participaram das duas intervenções em sequência e nesta mesma ordem. Primeiramente, foi aplicada a TENS, mas por doze minutos, sendo dois minutos de adaptação ao estímulo e dez minutos de estimulação. A Terapia Manual Laríngea foi aplicada em seguida, também por doze minutos. Foi realizada a aplicação de três minutos de massagem nos músculos esternocleidomastóideos; três minutos de massagem na região suprahióidea; um minuto e trinta segundos de aplicação de movimento de deslizamento e abaixamento na região da laringe; e um minuto e trinta segundos de aplicação de movimento de deslocamento lateral da região da tireoide; repetição de três minutos de massagem nos músculos esternocleidomastóideos^(36)^.

### Análise estatística

As variáveis quantitativas foram descritas por média e desvio padrão/erro padrão e as categóricas por frequências absolutas e relativas. O teste de normalidade de Shapiro-Wilk foi aplicado para verificar a distribuição das variáveis.

Para comparar médias entre os grupos, a Análise de Variância (ANOVA) foi aplicada. Na comparação de proporções, o teste qui-quadrado de Pearson em conjunto com a análise dos resíduos ajustados foi utilizado.

A comparação entre os momentos e entre os grupos foi realizada pelo modelo de Equações de Estimativas Generalizadas (GEE) complementado pelo teste *Least Significant Difference* (LSD). O modelo linear foi utilizado para as variáveis com distribuição normal e o modelo *tweedie* com função logarítmica foi aplicado para as variáveis com distribuição assimétrica. Todos os resultados foram ajustados para as variáveis que apresentaram p<0,10 na análise de comparação de grupos (profissional da voz e diagnósticos laríngeos).

O nível de significância adotado foi de 5% (p≤0,05) e as análises foram realizadas no programa SPSS versão 27.0.

## RESULTADOS

De acordo com a Figura 1, foram incluídas 44 mulheres no estudo, dentre as quais, 28 finalizaram todas as etapas do estudo. As participantes foram distribuídas nos grupos conforme a randomização realizada na pesquisa.

**Figura 1:** Fluxograma de participantes da pesquisa

*Desistência: Participantes que interromperam o estudo antes da conclusão das intervenções programadas, independentemente do motivo

De acordo com a Tabela 1 de caracterização da amostra, não houve diferença estatisticamente significativa entre os grupos em relação à média de idade das participantes. Foi verificada diferença estatisticamente significativa entre os grupos quanto ao uso de voz profissional e de patologias laríngeas.

**Tabela 1:** Caracterização dos grupos estudados, de acordo com a idade, profissão e características laríngeas

**Variáveis**	**TENS (n=9)**	**TML (n=10)**	**TENS+TML (n=9)**	**P**
Idade (anos) – média ± DP	40,7 ± 8,1	41,9 ± 11,1	41,8 ± 12,0	0,963
Profissional da voz – n (%)				0,001
Sim	6 (66,7)	0 (0,0)	7 (77,8)^*^	
Não	3 (33,3)	10 (100)*	2 (22,2)	
Diagnóstico laríngeo – n(%)				0,099
Sem alteração	2 (22,2)	8 (80,0)	3 (33,3)	
Cisto vocal	1 (11,1)	2 (20,0)	1 (11,1)	
Nódulos vocais	3 (33,3)	0 (0,0)	3 (33,3)	
Fenda vocal	3 (33,3)	0 (0,0)	2 (22,2)	

* Associação estatisticamente significativa pelo teste dos resíduos ajustados a 5% de significância

Quanto à avaliação perceptivo-auditiva, conforme a Tabela 2, houve melhora do parâmetro de grau geral da qualidade vocal no grupo TML e no grupo TENS+TML. O grupo TENS não apresentou diferença estatisticamente significativa. Não foi constatada mudança estatisticamente significativa quanto ao parâmetro rugosidade nos grupos avaliados. Já no parâmetro de soprosidade, foi detectada diminuição estatisticamente significativa no grupo TENS. Houve aumento no grupo TENS+TML, constatada com diferença estatisticamente significativa na comparação entre grupos. Em relação à tensão vocal, apenas o grupo TENS+TML apresentou melhora estatisticamente significativa.

**Tabela 2:** Análise perceptivo-auditiva com comparação intragrupos (antes e após intervenções terapêuticas) e entre os grupos estudados: TENS, TML e TENS+TML ajustado para profissional da voz e diagnósticos laríngeos

**Variáveis**	**TENS**	**TML**	**TENS+TML**	**Valor de p**
	**Média ± EP**	**Média ± EP**	**Média ± EP**	
G				
Pré	1,94 ± 0,20	1,72 ± 0,16	1,63 ± 0,26	0,764
Pós	1,48 ± 0,18	1,30 ± 0,14	1,27 ± 0,15	0,406
Diferença Pré-Pós	0,45 (-0,03 a 0,94)	0,42 (0,10 a 0,74)	0,35 (0,02 a 0,69)	0,972
Valor de p	0,066	0,011	0,038	
R				
Pré	1,56 ± 0,17	1,44 ± 0,12	1,68 ± 0,25	0,416
Pós	1,26 ± 0,17	1,24 ± 0,08	1,19 ± 0,18	0,754
Diferença Pré-Pós	0,29 (-0,22 a 0,81)	0,20 (-0,05 a 0,46)	0,50 (-0,04 a 1,03)	0,640
Valor de p	0,264	0,113	0,068	
B				
Pré	1,23 ± 0,41	0,45 ± 0,24	0,22 ± 0,14	0,083
Pós	0,46 ± 0,19^ab^	0,10 ± 0,09^b^	0,66 ± 0,22^a^	0,015
Diferença Pré-Pós	0,77 (0,14 a 1,41)^b^	0,35 (-0,11 a 0,81)^b^	-0,44 (-0,88 a 0,00)^a^	0,007
Valor de p	0,017	0,134	0,051	
S				
Pré	1,36 ± 0,17	1,50 ± 0,19	1,33 ± 0,16	0,857
Pós	1,14 ± 0,19	1,30 ± 0,17	0,89 ± 0,19	0,353
Diferença Pré-Pós	0,22 (-0,20 a 0,64)	0,20 (-0,05 a 0,46)	0,46 (0,12 a 0,79)	0,433
Valor de p	0,310	0,112	0,007	

a,b,c Letras iguais não diferem pelo teste *Least Significant Difference* (LSD) a 5% de significância

A Tabela 3 demonstrou aumento estatisticamente significativo dos Tempos Máximos de Fonação (TMF) após intervenção, em todos os grupos avaliados, não foi verificada diferença estatisticamente significativa entre eles. Na Escala de Sintomas Vocais (ESV) e no protocolo de Índice de Fadiga Vocal (IFV), todos os grupos reduziram significativamente seus escores após intervenção. No entanto, não foi detectada diferença estatisticamente significativa entre eles. O Índice S/Z não apresentou mudança estatisticamente significativa.

**Tabela 3:** Comparação de medidas aerodinâmicas e de sintomas vocais intragrupos (antes e após intervenções terapêuticas) e entre os grupos estudados: TENS, TML e TENS+TML ajustado para profissional da voz e diagnósticos laríngeos

**Variáveis**	**TENS**	**TML**	**TENS+TML**	Valor de p
	**Média ± EP**	**Média ± EP**	**Média ± EP**	
TMF total				
Pré	7,81 ± 0,73	9,51 ± 1,39	8,96 ± 1,12	0,205
Pós	10,8 ± 1,38	11,9 ± 1,56	11,4 ± 0,77	0,946
Diferença Pré-Pós	-2,95 (-5,19 a -0,71)	-2,42 (-3,95 a-0,89)	-2,41 (-4,16 a -0,66)	0,918
Valor de p	0,010	0,002	0,007	
Índice S/Z				
Pré	1,12 ± 0,08	1,00 ± 0,05	1,14 ± 0,09	0,410
Pós	0,99 ± 0,08	1,07 ± 0,08	1,05 ± 0,08	0,808
Diferença Pré-Pós	0,13 (-0,03 a 0,29)	-0,04 (-0,26 a 0,18)	0,09 (-0,13 a 0,31)	0,120
Valor de p	0,112	0,696	0,416	
ESV				
Pré	58,0 ± 8,38	55,8 ± 7,06	54,8 ± 3,66	0,629
Pós	26,6 ± 5,76	20,3 ± 4,69	28,2 ± 4,81	0,579
Diferença Pré-Pós	31,4 (12,1 a 50,8)	35,5 (20,5 a 50,5)	26,6 (19,1 a 34,0)	0,553
Valor de p	0,001	<0,001	<0,001	
IFV				
Pré	45,5 ± 5,01	48,1 ± 3,98	44,5 ± 2,23	0,940
Pós	23,9 ± 4,98	23,5 ± 3,88	24,2 ± 2,92	0,721
Diferença Pré-Pós	21,6 (10,9 a 32,2)	24,6 (16,2 a 33,0)	20,3 (14,4 a 26,2)	0,718
Valor de p	<0,001	<0,001	<0,001	

**Legenda:** TMF = Tempo Máximo de Fonação; TML = Terapia Manual Laríngea; ESV = Escala de Sintomas Vocais; IFV = Índice de Fadiga Vocal

Em relação à intensidade de dor musculoesquelética autorrelatada pelas participantes, conforme a Tabela 4, as regiões avaliadas de parte superior das costas, parte inferior das costas, músculo masseter e parte posterior do pescoço tiveram diminuição estatisticamente significativa, após as intervenções, em todos os grupos. Na região submandibular, os grupos TENS e TML não apresentaram mudança estatisticamente significativa. Já na região da frente do pescoço, o grupo TML não teve mudança na análise. Na região da laringe, foi constatada melhora nos grupos TML e TENS+TML com diferença estatisticamente significativa quando comparadas ao grupo TENS.

**Tabela 4:** Comparação da dor musculoesquelética nas diferentes regiões corporais intragrupos (antes e após intervenções terapêuticas) e entre os grupos estudados: TENS, TML e TENS+TML ajustado para profissional da voz e diagnósticos laríngeos

**Regiões Corporais**	**TENS**	**TML**	**TENS+TML**	Valor de p
	**Média ± EP**	**Média ± EP**	**Média ± EP**	
Superior das costas				
Pré	59,3 ± 8,96	50,9 ± 11,9	53,8 ± 10,8	0,609
Pós	20,5 ± 7,56	17,9 ± 6,46	15,1 ± 4,47	0,773
Diferença Pré-Pós	38,8 (15,3 a 62,4)	33,0 (9,83 a 56,2)	38,7 (15,3 a 62,1)	0,895
Valor de p	0,001	0,005	0,001	
Inferior das costas				
Pré	40,0 ± 9,18	48,2 ± 14,0	53,8 ± 10,8	0,319
Pós	27,4 ± 9,57	16,2 ± 8,70	19,0 ± 5,94	0,255
Diferença Pré-Pós	12,6 (1,51 a 23,7)	32,1 (9,74 a 54,4)	34,9 (12,6 a 57,1)	0,131
Valor de p	0,026	0,005	0,002	
Temporal				
Pré	39,9 ± 16,2	61,8 ± 25,3	17,4 ± 5,62	0,061
Pós	11,7 ± 4,52	5,76 ± 4,29	7,13 ± 4,92	0,756
Diferença Pré-Pós	28,2 (-1,80 a 58,2)	56,0 (8,21 a 103,8)	10,3 (-5,24 a 25,8)	0,323
Valor de p	0,065	0,022	0,194	
Masseter				
Pré	25,4 ± 7,78	45,6 ± 18,8	35,2 ± 8,93	0,200
Pós	11,3 ± 5,12	12,8 ± 7,69	13,2 ± 5,48	0,608
Diferença Pré-Pós	14,1 (4,42 a 23,7)	32,7 (2,19 a 63,3)	22,0 (0,54 a 43,5)	0,714
Valor de p	0,004	0,036	0,044	
Submandibular				
Pré	30,4 ± 8,77	19,3 ± 8,64	35,0 ± 12,2	0,828
Pós	10,6 ± 5,22	6,76 ± 5,53	9,76 ± 4,82	0,697
Diferença Pré-Pós	19,8 (-1,01 a 40,6)	12,5 (-7,17 a 32,2)	25,3 (1,22 a 49,3)	0,954
Valor de p	0,062	0,213	0,039	
Laringe				
Pré	31,4 ± 11,7	39,3 ± 12,8	51,5 ± 11,5	0,720
Pós	19,5 ± 6,17	1,88 ± 1,35	12,0 ± 9,09	0,292
Diferença Pré-Pós	11,9 (-17,5 a 41,3)^a^	37,4 (12,9 a 62,0)^b^	39,5 (10,8 a 68,2)^b^	0,019
Valor de p	0,428	0,003	0,007	
Frente do pescoço				
Pré	32,5 ± 12,2	18,4 ± 8,82	42,6 ± 11,9	0,578
Pós	4,36 ± 3,31	1,97 ± 1,68	11,5 ± 7,61	0,417
Diferença Pré-Pós	28,1 (5,43 a 50,8)	16,4 (-0,60 a 33,5)	31,1 (2,96 a 59,3)	0,684
Valor de p	0,015	0,059	0,030	
Posterior pescoço				
Pré	55,1 ± 8,81	60,7 ± 15,1	51,8 ± 10,2	0,581
Pós	18,3 ± 5,89	22,6 ± 6,82	16,2 ± 5,93	0,781
Diferença Pré-Pós	36,8 (16,9 a 56,7)	38,2 (10,7 a 65,7)	35,6 (13,7 a 57,5)	0,938
Valor de p	<0,001	0,007	0,001	

a,b,c Letras iguais não diferem pelo teste *Least Significant Difference* (LSD) a 5% de significância

Na tabela 5, quando analisados os níveis de sensação de tensão muscular à palpação, foi observada redução em todos os grupos avaliados no escore total. Durante a palpação em repouso, foi observada em região de submento uma diminuição da sensação de tensão muscular nos grupos TENS e TML; diminuição em região infrahióidea à esquerda; diminuição em região infrahióidea à direita exceto no grupo TENS+TML; cricotireoideo à esquerda apresentou diminuição exceto no grupo TENS; redução no cricotireoideo à direita em todos os grupos com constatação de diferença estatisticamente significativa entre os grupos; região do músculo esternocleidomastoideo apresentou redução à esquerda e à direita exceto no grupo TML. Quando analisados os níveis de sensação de tensão muscular durante a palpação à fonação, foi constatada redução em região do submento em todos os grupos; região infrahióideia à esquerda e à direita com redução nos grupos TML e TENS+TML e diferença estatisticamente significativa entre os grupos; na região do cricotireoideo à esquerda com redução significativa na comparação entre os grupos; na região do cricotireoideo à direita observou-se melhora de todos os grupos; na região do músculo esternocleidomastoideo esquerdo todos os grupos obtiveram melhora e observou-se diferença estatisticamente significativa entre os grupos; na região do músculo esternocleidomastoideo direito não foi observada redução no grupo TENS e foi constatada diferença significativa na comparação do grupo TENS com os outros dois grupos.

**Tabela 5:** Comparação do nível de sensação de tensão muscular de diferentes regiões corporais intragrupos (antes e após intervenções terapêuticas) e entre os grupos estudados: TENS, TML e TENS+TML ajustado para profissional da voz e diagnósticos laríngeos

**Regiões**	**TENS**	**TML**	**TENS+TML**	**Valor de p**	
	**Média ± EP**	**Média ± EP**	**Média ± EP**		
Total					
Pré	61,5 ± 10,0	53,0 ± 7,31	60,0 ± 7,77	0,609	
Pós	29,2 ± 6,56	14,9 ± 2,40	14,4 ± 2,22	0,052	
Diferença Pré-Pós	32,3 (23,0 a 41,6)^a^	38,1 (26,8 a 49,4)^ab^	45,7 (32,9 a 58,4)^b^	<0,001	
Valor de p	<0,001	<0,001	<0,001		
P.R.Submento					
Pré	1,13 ± 0,33	0,84 ± 0,26	0,97 ± 0,31	0,858	
Pós	0,42 ± 0,21	0,22 ± 0,14	0,37 ± 0,19	0,714	
Diferença Pré-Pós	0,71 (0,09 a 1,33)	0,62 (0,13 a 1,12)	0,60 (0,00 a 1,20)	0,874	
Valor de p	0,024	0,013	0,051		
P.R.Infrahióidea (E)					
Pré	0,95 ± 0,32	0,40 ± 0,14	1,12 ± 0,46	0,734	
Pós	0,43 ± 0,21	0,11 ± 0,07	0,09 ± 0,08	0,377	
Diferença Pré-Pós	0,52 (0,13 a 0,92)	0,29 (0,02 a 0,56)	1,03 (0,08 a 1,98)	0,280	
Valor de p	0,010	0,038	0,034		
P.R.Infrahióidea (D					
Pré	0,92 ± 0,29	0,66 ± 0,27	0,95 ± 0,50	0,921	
Pós	0,52 ± 0,22	0,07 ± 0,07	0,09 ± 0,09	0,171	
Diferença Pré-Pós	0,40 (0,10 a 0,71)	0,59 (0,07 a 1,10)	0,86 (-0,15 a 1,87)	0,093	
Valor de p	0,009	0,026	0,096		
P.R.Cricotireoideo (E)					
Pré	0,89 ± 0,31	0,90 ± 0,24	1,42 ± 0,41	0,232	
Pós	0,41 ± 0,21	0,10 ± 0,10	0,00 ± 0,00	0,121	
Diferença Pré-Pós	0,48 (-0,28 a 1,24)	0,80 (0,32 a 1,28)	1,42 (0,61 a 2,24)	0,215	
Valor de p	0,218	0,001	<0,001		
P.R.Cricotireoideo (D)					
Pré	0,99 ± 0,27	0,91 ± 0,26	1,77 ± 0,38	0,056	
Pós	0,20 ± 0,12	0,00 ± 0,00	0,00 ± 0,00	0,266	
Diferença Pré-Pós	0,79 (0,21 a 1,37)^a^	0,91 (0,40 a 1,43)^ab^	1,77 (1,03 a 2,50)^b^	0,044	
Valor de p	0,008	<0,001	<0,001		
P.R. ECOM (E)					
Pré	1,06 ± 0,31	1,23 ± 0,62	0,75 ± 0,21	0,461	
Pós	0,45 ± 0,17^b^	0,09 ± 0,09^ab^	0,07 ± 0,07^a^	0,019	
Diferença Pré-Pós	0,61 (0,27 a 0,95)	1,14 (-0,04 a 2,31)	0,68 (0,23 a 1,12)	0,072	
Valor de p	<0,001	0,058	0,003		
P.R. ECOM (D)					
Pré	0,94 ± 0,31	1,18 ± 0,59	0,85 ± 0,29	0,941	
Pós	0,54 ± 0,21^b^	0,18 ± 0,12^ab^	0,07 ± 0,07^a^	0,010	
Diferença Pré-Pós	0,40 (0,02 a 0,79)	1,00 (-0,12 a 2,13)	0,77 (0,14 a 1,40)	0,066	
Valor de p	0,040	0,081	0,016		
P.F.Submento					
Pré	1,79 ± 0,46	1,25 ± 0,33	1,25 ± 0,28	0,605	
Pós	1,10 ± 0,34	0,34 ± 0,18	0,31 ± 0,14	0,118	
Diferença Pré-Pós	0,69 (0,10 a 1,29)	0,91 (0,39 a 1,43)	0,94 (0,33 a 1,54)	0,089	
Valor de p	0,022	<0,001	0,002		
P.F.Infrahióidea (E)					
Pré	1,13 ± 0,38	0,82 ± 0,30	1,32 ± 0,28		0,863
Pós	1,03 ± 0,37^b^	0,27 ± 0,13^ab^	0,09 ± 0,09^a^		0,024
Diferença Pré-Pós	0,11 (-0,09 a 0,30)^a^	0,55 (0,00 a 1,10)^ab^	1,23 (0,64 a 1,81)^b^		0,005
Valor de p	0,288	0,050	<0,001		
P.F.Infrahióidea (D)					
Pré	1,30 ± 0,33	1,09 ± 0,32	1,30 ± 0,30		0,880
Pós	1,08 ± 0,29^b^	0,17 ± 0,12^a^	0,20 ± 0,12^a^		0,001
Diferença Pré-Pós	0,22 (-0,06 a 0,50)^a^	0,92 (0,37 a 1,47)^b^	1,09 (0,48 a 1,71)^b^		0,001
Valor de p	0,126	0,001	<0,001		
P.F.Cricotireoideo (E)					
Pré	1,13 ± 0,35	1,96 ± 0,72	1,13 ± 0,29		0,711
Pós	0,58 ± 0,19^b^	0,30 ± 0,20^ab^	0,08 ± 0,07^a^		0,005
Diferença Pré-Pós	0,55 (0,08 a 1,02)^a^	1,66 (0,33 a 3,00)^ab^	1,06 (0,44 a 1,67)^b^		0,038
Valor de p	0,021	0,014	<0,001		
P.F.Cricotireoideo (D)					
Pré	1,34 ± 0,38	1,36 ± 0,53	1,53 ± 0,41		0,836
Pós	0,54 ± 0,20	0,00 ± 0,00	0,19 ± 0,12		0,067
Diferença Pré-Pós	0,79 (0,34 a 1,25)	1,36 (0,32 a 2,41)	1,34 (0,52 a 2,17)		0,092
Valor de p	<0,001	0,010	0,001		
P.F. ECOM (E)					
Pré	1,38 ± 0,44	1,47 ± 0,54	1,58 ± 0,33		0,766
Pós	0,63 ± 0,21^b^	0,10 ± 0,10^a^	0,10 ± 0,09^a^		0,048
Diferença Pré-Pós	0,75 (0,16 a 1,33)^a^	1,37 (0,35 a 2,39)^b^	1,48 (0,81 a 2,16)^b^		0,020
Valor de p	0,012	0,009	<0,001		
P.F. ECOM (D)					
Pré	1,18 ± 0,37	2,16 ± 0,86	1,21 ± 0,27		0,691
Pós	0,65 ± 0,20^b^	0,12 ± 0,11^a^	0,07 ± 0,06^a^		0,003
Diferença Pré-Pós	0,52 (0,00 a 1,05)^a^	2,05 (0,40 a 3,70)^b^	1,14 (0,59 a 1,69)^b^		0,006
Valor de p	0,052	0,015	<0,001		
Posição alta da laringe					
Pré	1,55 ± 0,25^b^	1,69 ± 0,38^ab^	1,00 ± 0,20^a^		0,031
Pós	0,57 ± 0,14	0,53 ± 0,17	0,36 ± 0,13		0,171
Diferença Pré-Pós	0,98 (0,56 a 1,40)	1,17 (0,31 a 2,02)	0,63 (0,12 a 1,14)		0,938
Valor de p	<0,001	0,007	0,016		
Lateralização da laringe					
Pré	1,82 ± 0,22	2,12 ± 0,41	1,66 ± 0,28		0,917
Pós	0,85 ± 0,25	0,72 ± 0,22	0,65 ± 0,16		0,576
Diferença Pré-Pós	0,97 (0,54 a 1,39)	1,40 (0,68 a 2,13)	1,01 (0,62 a 1,40)		0,671
Valor de p	<0,001	<0,001	<0,001		

a,b,c Letras iguais não diferem pelo teste *Least Significant Difference* (LSD) a 5% de significância

**Legenda:** P.R. = Palpação em repouso; P.F. = Palpação durante a fonação; ECOM = músculo esternocleidomastóideo

Foram consideradas para análise apenas palpação com toque suave “*tenderness*”

Nos parâmetros de posição alta da laringe no pescoço e resistência à lateralização laríngea, houve diminuição significativa em todos os grupos após as intervenções, sem diferença entre eles.

## DISCUSSÃO

As técnicas aplicadas nesta pesquisa têm sido analisadas como possíveis recursos na reabilitação de indivíduos com disfonia associada à tensão muscular^(15-21,37)^. Este estudo comparou as técnicas isoladamente e associadas para verificar seus efeitos em relação aos parâmetros de qualidade vocal, autopercepção vocal e de dor musculoesquelética, medidas aerodinâmicas e níveis de tensão muscular.

Quanto à caracterização da amostra e dos grupos, houve uma predominância de participantes de meia-idade, profissionais da voz e que apresentavam alteração laríngea de origem funcional e organofuncional, o que condiz com o perfil de participantes com DTM^(3-4)^. No grupo TML, foram observadas diferenças específicas quanto ao uso profissional da voz e às alterações laríngeas, considerando que a amostra foi predominantemente composta por indivíduos não profissionais da voz e, em sua maioria, sem alterações laríngeas. Essa característica amostral foi criteriosamente controlada e considerada nas análises estatísticas, a fim de minimizar vieses e assegurar a validade dos resultados. As mulheres nesta faixa etária e que fazem uso profissional da voz são mais suscetíveis a desenvolverem disfonia, pois apresentam fatores biológicos e comportamentais que favorecem o desequilíbrio do funcionamento muscular laríngeo^(38)^. Existe uma diferença de proporção entre a laringe feminina e masculina, assim como as pregas vocais femininas são mais curtas e menos espessas, o que aumenta o risco de colisões e a dificuldade de absorção de lesões. Também há uma distribuição assimétrica de ácido hialurônico na lâmina própria das pregas vocais femininas, o que dificulta o fechamento glótico^(39-41)^. As mulheres também são mais suscetíveis aos fatores psicossociais que originam problemas emocionais^(1)^. Isto aumenta o risco de desenvolverem estresse e ansiedade, originando tensão muscular excessiva e quadros de dores musculoesqueléticas crônicas^(33)^. Quando estas mulheres fazem uso profissional da voz, o risco de desenvolverem disfonia aumenta, pois muitas delas não possuem conhecimento adequado acerca de saúde vocal e apresentam comportamento vocal inadequado^(41)^.

Este estudo demonstrou os efeitos positivos das duas técnicas, aplicadas de forma isolada e combinadas, em relação aos sintomas vocais e de fadiga vocal. As mulheres referiram melhora em relação aos seus sintomas vocais, o que afeta positivamente o seu cotidiano de maneira funcional, física e emocional. Estudos prévios apontam que o uso de ambas as técnicas utilizadas nesta pesquisa possuem efeitos positivos nos sintomas autorreferidos dos participantes^(18,21)^. Além disso, houve diminuição significativa de sintomas de fadiga vocal, referidas pelas mulheres do estudo. A fadiga vocal pode acometer participantes com DTM, pois têm relação com ajustes vocais e musculares inadequados e com sensação de esforço ao falar^(42)^.

Quanto à autopercepção de dor musculoesquelética, os três grupos apresentaram respostas positivas quanto à diminuição de dor autorreferida em região perilaríngea e cervical. Estes resultados corroboram com os estudos que demonstraram diminuição de sintoma de dor em região perilaríngea e adjacentes com o uso de TENS e TML^(13,18,22)^. Quanto às estruturas avaliadas, destaca-se a melhora na região da laringe referidas pelas participantes dos grupos TML e TENS+TML, que receberam a TML em região laríngea, em comparação ao grupo TENS, que recebeu a aplicação da TENS de forma isolada. É possível concluir que, por fazer uso de uma manipulação direta na musculatura extrínseca da laringe, a TML foi mais eficaz na redução de sensação de dor nesta região^(11)^. Diante dos resultados encontrados, infere-se que a combinação da técnica de eletroterapia associada à terapia manual laríngea demonstram mais resultados positivos na região do pescoço e no arcabouço laríngeo. Isto porque a aplicação das técnicas associadas proporcionou maior sensação de relaxamento e de conforto muscular na região perilaríngea e cervical^(10-11)^.

Destaca-se o aumento dos Tempos Máximos de Fonação (TMF) nos três grupos avaliados. O TMF é um parâmetro clínico que indica a capacidade de controle das forças aerodinâmicas e mioelásticas da laringe^(43)^. O aumento significativo verificado no presente estudo pode estar relacionado com uma diminuição da ação mioelástica no equilíbrio entre as duas forças, o que pode ter proporcionado melhora do funcionamento muscular e respiratório durante a fonação^(44)^. Já a relação s/z demonstrou normalidade antes das técnicas aplicadas e se manteve assim após a terapia, o que pode indicar que a medida não foi capaz de indicar o padrão hiperfuncional das participantes avaliadas.

Quanto à avaliação de qualidade vocal, foi possível verificar melhora do grau geral de desvio vocal nos grupos que receberam TML isolada (TML) ou associada à TENS (TENS+TML). Pode-se inferir que o uso da TML teve melhor efeito no equilíbrio fonatório, melhorando a qualidade vocal das mulheres nestes dois grupos. Este resultado difere de outro estudo^(21)^, que encontrou melhores efeitos imediatos na qualidade vocal em mulheres com disfonia comportamental que utilizaram a TENS, em comparação às que receberam apenas TML. Destaca-se a relação entre os parâmetros de tensão vocal e soprosidade. Uma relação semelhante foi encontrada em outro estudo^(24)^ com a aplicação de TENS de baixa frequência associada aos exercícios vocais. Nos grupos TENS e TML houve pouca redução de tensão vocal. Já no grupo TENS+TML, pela significativa redução de tensão é possível evidenciar um aumento de soprosidade, o que pode ter ocorrido por um provável ajuste da laringe. Outra pesquisa^(45)^ aplicou TML em participantes com DTM primária e concluiu que medidas acústicas e de fechamento glótico para vários participantes revelaram insuficiência de fechamento glótico após o tratamento, quando a hiperfunção foi diminuída com a TML. Os autores concluem que, para estes participantes, é importante a aplicação de técnicas complementares para auxiliar na função de fechamento glótico equilibrado.

Quanto aos níveis de sensação de tensão muscular à palpação, os grupos analisados apresentaram melhora significativa após as intervenções realizadas, com destaque para o grupo que utilizou as técnicas de eletroterapia associada à terapia manual laríngea. As técnicas aplicadas foram eficazes na diminuição da sensação de tensão muscular na musculatura extrínseca da laringe à palpação e em estruturas adjacentes, pois têm como efeito principal o aumento da impressão de relaxamento muscular destas estruturas^(13,18)^. O grupo TENS+TML, por associar as duas técnicas, teve melhor efeito de diminuição de sensação de tensão muscular à palpação nesta região, principalmente quando avaliada durante a fonação. As técnicas associadas promoveram com maior precisão a sensação de redução de tensão da musculatura extrínseca da laringe, o que pode ter sido ocasionado por uma diminuição do acionamento do sistema de compensação muscular durante a fonação das participantes. Isso pode ter repercutido positivamente no funcionamento deste agrupamento de músculos.

Entre as limitações deste estudo, destaca-se a ausência de um grupo controle, o que restringiu as possibilidades de comparação dos resultados obtidos. Observou-se, ainda, uma discrepância entre os grupos quanto ao uso profissional da voz e à presença de alterações vocais, fator considerado nas análises estatísticas. Como a alocação dos participantes foi realizada por randomização, esperava-se que os grupos apresentassem características homogêneas. No entanto, em estudos com tamanho amostral limitado, diferenças entre grupos podem ocorrer por efeito do acaso. Para minimizar esse potencial viés, foi realizada a análise multivariada ajustando para as variáveis “profissional da voz” e “diagnóstico laríngeo”, com o objetivo de tornar os grupos mais comparáveis e garantir maior validade na interpretação dos resultados. Além disso, não foi possível realizar a análise acústica da voz, nem o exame de imagem laríngea após as intervenções, devido à ausência de infraestrutura laboratorial adequada para garantir o rigor metodológico necessário. Diante disso, recomenda-se que pesquisas futuras incluam a análise acústica da voz e exames de imagem laríngea para aprofundar a compreensão dos efeitos das técnicas aplicadas. Sugere-se, ainda, a utilização de avaliações complementares, como a eletromiografia de superfície, para investigar de forma objetiva o impacto das intervenções na musculatura perilaríngea.

## CONCLUSÃO

Os achados deste estudo demonstram que as intervenções propostas exerceram efeitos terapêuticos relevantes em mulheres com Disfonia por Tensão Muscular. Houve redução de sintomas vocais, diminuição da fadiga vocal, redução de dor musculoesquelética e aumento dos tempos máximos de fonação nos três grupos avaliados. Quanto à qualidade vocal, as técnicas TENS e TML isoladas diminuíram, respectivamente, os graus de soprosidade e grau geral da disfonia. No entanto, apenas quando aplicadas de forma combinada, foram capazes de diminuir o grau geral da disfonia e o grau de tensão vocal, proporcionando melhor ajuste vocal, já que a tensão vocal é uma importante característica da população estudada. Quanto à tensão muscular, apenas a TML isolada ou combinada com a TENS foi capaz de reduzir a sensação de tensão muscular à palpação. Portanto, as técnicas combinadas foram capazes de proporcionar melhores ajustes vocais e diminuir a sensação de tensão muscular à palpação.
